# Inherent Ethyl Acetate Selectivity in a Trianglimine Molecular Solid

**DOI:** 10.1002/chem.202101510

**Published:** 2021-05-27

**Authors:** Donglin He, Chengxi Zhao, Linjiang Chen, Marc A. Little, Samantha Y. Chong, Rob Clowes, Katherine McKie, Mark G. Roper, Graeme M. Day, Ming Liu, Andrew I. Cooper

**Affiliations:** ^1^ Department of Chemistry and Materials Innovation Factory University of Liverpool Liverpool L7 3NY UK; ^2^ Key Laboratory for Advanced Materials and School of Chemistry and Molecular Engineering East China University of Science and Technology Shanghai 200237 China; ^3^ Leverhulme Research Centre for Functional Materials Design University of Liverpool Liverpool L7 3NY UK; ^4^ Hiden Isochema Ltd. Warrington WA5 7TS UK; ^5^ Computational Systems Chemistry School of Chemistry University of Southampton Southampton SO17 1BJ UK

**Keywords:** crystal structure prediction, dynamic separation, macrocycles, molecular crystals, selective adsorption

## Abstract

Ethyl acetate is an important chemical raw material and solvent. It is also a key volatile organic compound in the brewing industry and a marker for lung cancer. Materials that are highly selective toward ethyl acetate are needed for its separation and detection. Here, we report a trianglimine macrocycle (**TAMC**) that selectively adsorbs ethyl acetate by forming a solvate. Crystal structure prediction showed this to be the lowest energy solvate structure available. This solvate leaves a metastable, “templated” cavity after solvent removal. Adsorption and breakthrough experiments confirmed that **TAMC** has adequate adsorption kinetics to separate ethyl acetate from azeotropic mixtures with ethanol, which is a challenging and energy‐intensive industrial separation.

## Graphical abstract

**The separation** of ethyl acetate from its azeotropic mixtures with ethanol is of great importance in the industrial production of ethyl acetate. Current purification techniques include extractive distillation and azeotropic distillation are energy‐intensive. Guided by crystal structure prediction, a “templating” strategy was used to construct selective binding sites in a trianglimine macrocycle crystal for the solvent molecule. These crystals exhibit inherently high selectivity towards ethyl acetate, reasonable kinetics, and show promise for real‐life, practical dynamic separations.

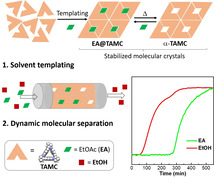

## Introduction

Ethyl acetate (EA) is an key solvent for the chemical industry; it is also a key volatile organic compound (VOC) that can determine the flavor and quality of beers or wines.^[1]^ EA is also a biomarker for the early diagnosis of lung cancer.^[2]^ Fisher esterification of ethanol (EtOH) is the main industrial process for synthesizing EA. The subsequent separation of EA from EtOH is difficult because they have similar boiling points (78.5 °C and 77.1 °C, respectively); also, EA and EtOH form azeotropic mixtures.^[3]^ Current purification techniques include extractive distillation,^[4]^ azeotropic distillation using ionic liquids,^[5]^ and membrane separation,^[6]^ but these can be inefficient and energy‐intensive. There is potential to find alternative separation processes based on selective adsorption using microporous materials that can operate under atmospheric conditions. In addition to separations, porous materials with high selectivity for EA might be used as sorbents in thermal desorption techniques^[7]^ to quantify trace level (ppm) EA from a gas mixture.

Organic macrocycles have been widely studied in solution as hosts for various guest molecules, including organic molecules, metal cations, and nucleotides.^[8]^ Macrocycles have also shown potential as solid‐state porous media for the selective adsorption of gases or vapors. For example, β‐cyclodextrin (β‐CD) was shown to capture VOCs such as styrene, aniline, and benzaldehyde from polluted air.^[9]^ Calix‐[4]‐arene macrocycles with hydrophobic cavities were reported to adsorb VOCs such as toluene, benzene, nitrobenzene and phenol selectively from aqueous solution.^[10]^ We showed that pillar[n]arenes can be used to separate styrene from ethylbenzene and to adsorb para‐xylene from its structural isomers.^[11]^

**Figure 1:**
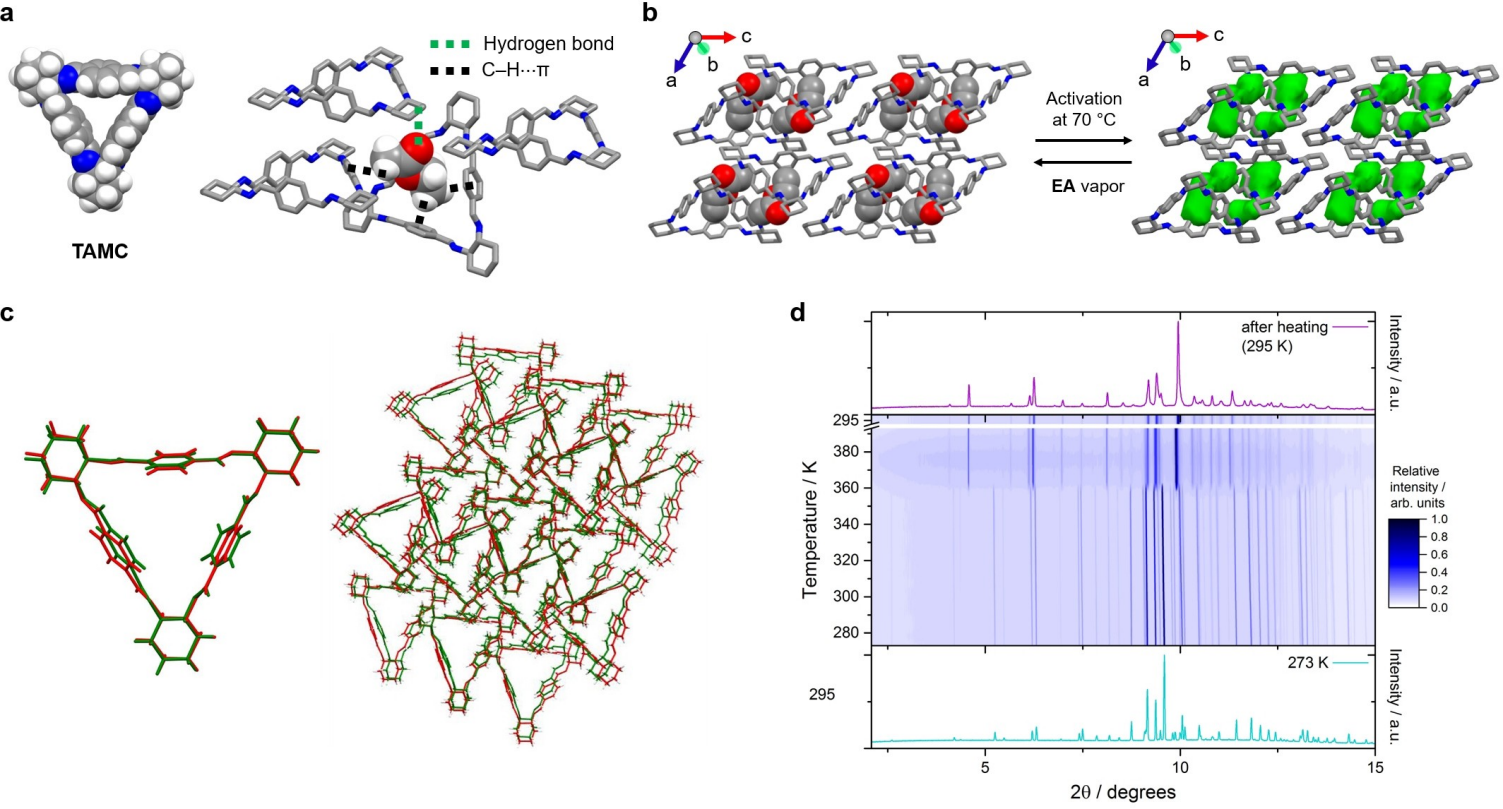
Single‐crystal structures: (a) EA@**TAMC** showing hydrogen bonding and C−H⋅⋅⋅π interactions between **TAMC** and EA; (b) crystal packings for EA@**TAMC** (left) and *α*‐**TAMC** (right) with EA‐selective voids colored in green. Hydrogen atoms are omitted for clarity. (c) Crystal packing overlay for EA@**TAMC** (green) and *α*‐**TAMC** (red), as generated using the crystal packing similarity tool in *Mercury*. (d) Powder X‐ray diffraction data collected during in situ heating (273–393 K, then cooled to 295 K) for a sample of EA@**TAMC** contained in a borosilicate glass capillary (diameter=0.5 mm).

The trianglimine macrocycle (**TAMC**) shown in Figure 1a is formed from the condensation of terephthalaldehyde and trans‐1,2‐cyclohexanediamine, and was first reported in 2000.^[12]^ The amine form of **TAMC** was reported to be a good host for tricarboxylic acids or anions in solution.^[13]^ Most recently, **TAMC** was shown to form supramolecular organic frameworks with intrinsic porosity, which show good selectivity for CO_2_ over CH_4_.^[14]^ Here, we show that EA is accommodated in the **TAMC** cavity to form a 1 : 1 solvate, EA@**TAMC**, which forms near‐perfect selective binding pockets for the EA guest. EA@**TAMC** adopts the lowest energy packing arrangement, as calculated by crystal structure prediction for a 1 : 1 mixture of **TAMC** and EA molecules. Crystal structure prediction (CSP) also finds that the same packing mode when EA is removed corresponds to a stable, albeit high energy, structure on the predicted landscape of **TAMC**. The guest‐free structure, *α*‐**TAMC**, retains these near‐perfect binding pockets after solvent removal to re‐adsorb EA. The specificity of these binding sites in *α*‐**TAMC** for EA over EtOH drives the high selectivity that we observed in competitive adsorption breakthrough measurements, where *α*‐**TAMC** can separate EA from an EA‐EtOH vapor mixture in one cycle under ambient temperature and pressure.

## Results and Discussion

### Structural analysis of TAMC

**TAMC** can be purified by recrystallization from EA during synthesis.^[12]^ Unexpectedly, we found that one molecule of EA per **TAMC** molecule remained in the structure after the **TAMC** crystals were activated under a high vacuum at room temperature, in which condition EA is normally expected to be easily removed; this was deduced initially from thermogravimetric analysis (TGA, Figure S1) and ^1^H NMR data (Figure S2). We found that these residual EA molecules were bound strongly, and they could only be removed from the solvated crystals by heating at temperatures above 70 °C under a high vacuum for about 12 h (Figure S4). The single crystal structure for EA@**TAMC** confirmed the 1 : 1 **TAMC**: EA molar ratio, with the ethyl group of each EA molecule being located in the center of the **TAMC** cavity, interacting with **TAMC** via three of C−H⋅⋅⋅π interactions (Figure 1a).^[12]^ The acetate ester group of EA is located in an extrinsic void created between three **TAMC** molecules. There is also a hydrogen bond interaction between the carbonyl oxygen atom of EA and the cyclohexane group of a second **TAMC** molecule.

After thermally removing the EA solvent from EA@**TAMC** at 70 °C, we found that the structure of the activated crystals, *α*‐**TAMC**, was closely related to EA@**TAMC**, based on similarities between their single crystal structures (Figure 1b) and powder X‐ray diffraction (PXRD) patterns (Figure S5a). After activation, the unit cell volume decreased by 5.4 %, which is mainly due to a structural contraction of 0.5 Å along the b‐axis (Figure 1b). However, the packing of **TAMC** molecules in EA@**TAMC** and desolvated *α*‐**TAMC** is essentially isostructural (Figure 1b–c, Supporting Information, Figure S6, Table S2; CCDC numbers 2049238 and 2049237 ), and hence near‐perfect voids for adsorbing EA are retained in *α*‐**TAMC**. PXRD data collected during in situ heating (273–393 K, then cooled to 295 K) for EA@**TAMC** (Figure 1d) show that the bulk material also undergoes limited rearrangement when the solvent is removed. The unit cell (Table S2) indicates a reduced symmetry structure with lattice dimensions and molecular volume for the macrocycle close to that of EA@**TAMC**.

### Vapor‐phase adsorption studies

Inspired by the potentially EA‐selective voids in *α*‐**TAMC**, we first investigated the adsorption of EA as a single, pure component. After being exposed to EA vapor, *α*‐**TAMC** adsorbs EA at 1 : 1 molar ratio, as confirmed by NMR (Figure S9), which agrees with the molar ratio observed in the EA@**TAMC** crystal structure. Furthermore, the PXRD patterns of *α*‐**TAMC** after exposure to both EA vapor and EA‐EtOH vapor mixtures yield PXRD patterns that are very similar to EA@**TAMC** (Figure S11), suggesting that the EA@**TAMC** crystal is formed both in EA vapor and in EA‐EtOH vapor mixtures.

**Figure 2:**
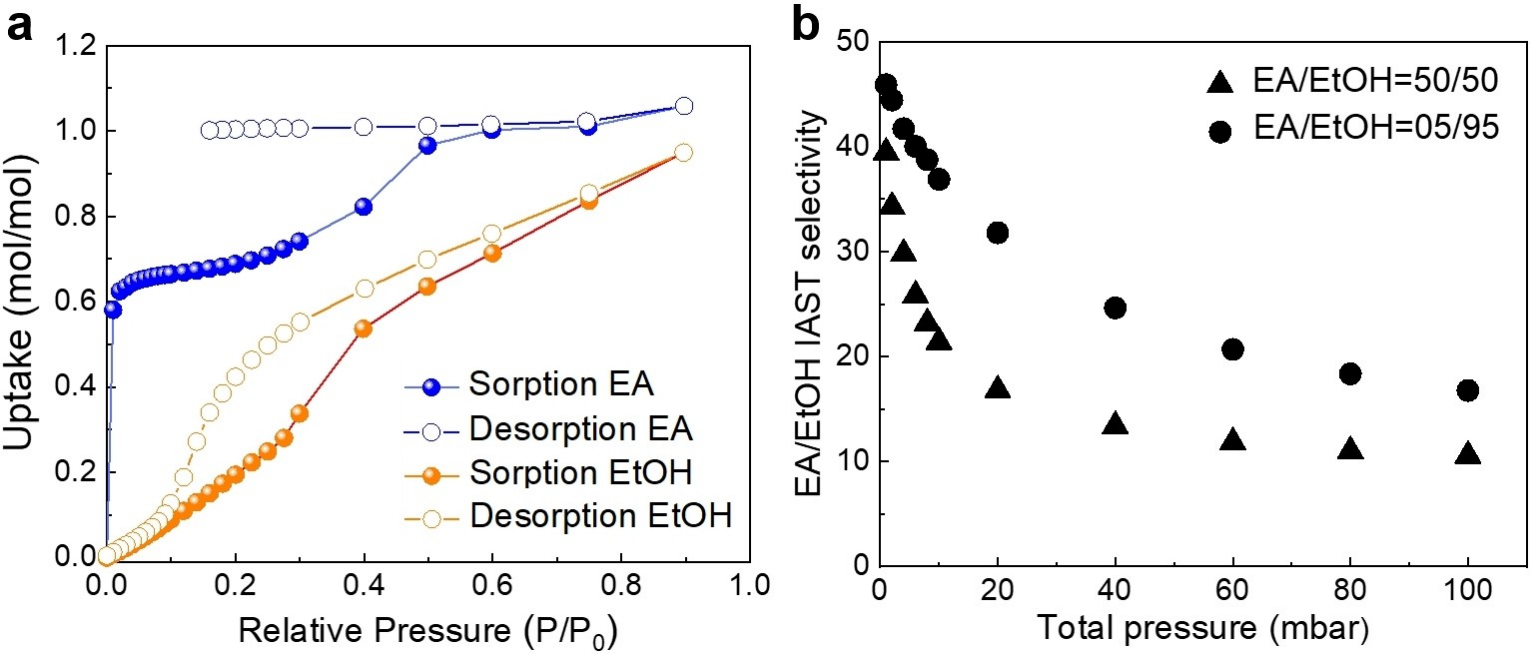
(a) EtOH (P_0_=7.95 kPa) and EA (P_0_=13.33 kPa) vapor sorption isotherms for *α*‐**TAMC** at 298 K. (b) IAST selectivity of EA‐EtOH mixtures for *α*‐**TAMC** at 298 K as calculated from these pure component vapor sorption isotherms.

The single‐component EtOH and EA vapor isotherms for *α*‐**TAMC** were measured by gravimetric sorption apparatus (IGA‐002, Hiden Isochma) at 25 °C (Figure 2a) based on the change in sample mass as a function of pressure. In the EA isotherm, a steep rise was observed in uptake in the low relative pressure region (P/P_0_<0.00975). A second, less steep rise was then observed in the relative pressure range 0.01–0.3, before the isotherm reached the expected 1 mol/mol saturation point at P/P_0_ ∼0.5. The desorption isotherm for EA shows that the material is still saturated with EA at P/P_0_ at 0.15. This hysteresis behavior can be attributed to the strong adsorbent‐adsorbate interactions in EA@**TAMC**. By contrast, the EtOH adsorption isotherm is completely different: there is a linear uptake in the low relative pressure region (Figure 2a). The EtOH desorption isotherm also shows some hysteresis, but it is far less pronounced than for EA. The single‐component isotherms indicate that *α*‐**TAMC** has a stronger affinity for EA than EtOH, and the EA uptake is much higher than for EtOH in the low relative pressure region (P/P_0_<0.3).

The EA‐EtOH binary mixture adsorption selectivity was then predicted using ideal adsorption solution theory (IAST) based on the single‐component isotherms shown in Figure 2a (Supporting Information, section 8.5).^[15]^ The IAST‐predicted selectivity is shown in Figure 2b for binary mixtures of EA‐EtOH with compositions of 50 : 50 and 5 : 95 at 298 K. For equimolar mixtures, the initially predicted selectivity was 39.4, and then gradually decreased to 10.6 at 0.1 bar; this is much higher than the selectivity reported for ZIF‐8 (1.7–8.3) under the same conditions calculated by the same method.^[3]^ Even for the 5 : 95 EA‐EtOH binary mixture, EA is still predicted to be preferentially captured by **TAMC** (Figure 2b, solid dots).

**Figure 3:**
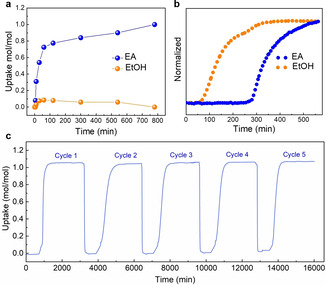
(a) Time‐dependent **TAMC** solid‐vapor sorption plot for a 1 : 1 v/v EA‐EtOH mixture vapor at 25 °C. (b) Breakthrough profiles for a 1 : 1 v/v EA‐EtOH vapor mixture at 25 °C (sorbate rate: 1.5 mL/min; total flow rates: 5 mL/min with a carrier gas of N_2_). (c) Adsorption‐desorption cycles for pure EA vapor (P_0_=13.33 kPa) at 25 °C (degas temperature at 70 °C) recorded by a gravimetric sorption analyzer.

To see whether *α*‐**TAMC** selectively adsorbs EA from actual EA‐EtOH vapor mixtures, we carried out time‐dependent static solid‐vapor sorption experiments using a EA‐EtOH (1 : 1 v/v) mixture at room temperature (Supporting Information, section 4.2). The uptake of EA and EtOH by *α*‐**TAMC** was determined by ^1^H NMR after dissolving the crystals in CDCl_3_. As shown in Figure 3a, the EA uptake increased sharply to ∼0.80 mol/**TAMC** after 120 mins. By contrast, the EtOH uptake was much lower (0.17 mol/**TAMC**) after the same time period. The EtOH uptake decreased slowly after this time, suggesting that adsorbed EtOH molecules might be replaced by EA molecules in the structure over time as the system equilibrates. Indeed, after 14 h exposure to the EA‐EtOH vapor mixture for 14 h, no EtOH could be detected in the ^1^H NMR spectrum of the dissolved crystals (Figure S12) and the EA uptake saturated at 1 mol/mol EA/**TAMC** (1.57 mmol/g), consistent with TGA, X‐ray diffraction, and vapor isotherm data. This suggests that the initial EtOH adsorption at shorter times is a kinetic process. Likewise, **TAMC** captures EA selectively from an EA‐EtOH azeotropic mixture vapor (30 : 70 wt.%, 72 °C), which is a mixture that is difficult to separate in the industrial Fisher esterification process. This was proven by further time‐dependent solid‐vapor sorption experiments (Supporting Information, Section 4.3, Figure S13). We also investigated the EA selectivity of *α*‐**TAMC** at low EA concentrations. When dry N_2_ containing EA (100–500 ppm, a range that EA can be detected in wine) and EtOH (200–800 ppm) was passed through a column packed with *α*‐**TAMC** at a flow rate of 0.2 L/min for 20 minutes, we found that only EA was adsorbed by *α*‐**TAMC**, as confirmed by NMR (Supporting Information, Section 4.5, Table S3).

### Dynamic separation of EA‐EtOH mixture using TAMC solid

To evaluate the performance of *α*‐**TAMC** for the dynamic separation of EA‐EtOH mixtures under flow, we performed breakthrough measurements using an adsorption column packed with activated *α*‐**TAMC** (Supporting Information, section 7). Breakthrough profiles for a 1 : 1 v/v EA‐EtOH vapor mixture at 25 °C are shown in Figure 3b. Both EA and EtOH were retained in the *α*‐**TAMC** column at the beginning of the experiment at a carrier gas flow rate of 1.5 mL/min. The EtOH then broke through in the period 50–300 min. By contrast, EA was retained until 300 min, and did not break through until almost all of the EtOH had eluted. Then EA signal then increased gradually until the feed concentration was reached after about 500 mins. This breakthrough experiment confirms the potential of *α*‐**TAMC** to cleanly separate EA and EtOH in real flow processes.

Repeated EA sorption‐desorption cycles were performed using a gravimetric sorption apparatus (Supporting Information, section 1.5.5). In contrast to many crystalline framework materials, which tend to lose performance because of a progressive decrease in crystallinity during repeat sorption cycles, there was no drop in the adsorption performance of the *α*‐**TAMC** material after 5 cycles (Figure 3c). This suggests that *α*‐**TAMC** could be suitable for actual separation applications in the future.

### Crystal structure prediction and electrostatic surface potential analysis

Different polymorphs of **TAMC** can be obtained using other solvents (Figures S17–19).^[16]^ These other polymorphs of **TAMC** can also selectively adsorb EA via a structural transformation process. For example, when **TAMC** was crystallized from dichloromethane and then exposed to EA or a mixed EA‐EtOH vapor, the structure gradually transformed into the EA solvate structure, EA@**TAMC**, although the dynamics of this transformation were slower, taking over 16 hours (Figures S20, S23). Similar experimental results were also found for a different polymorph induced by acetone (Figure S24). These results show that **TAMC** can selectively adsorbing EA by adaptation to EA@**TAMC** structure, even if we do not start with the *α*‐**TAMC** phase, albeit at the expense of slower kinetics.

Solvated molecular crystals often transform to another phase or collapse when the adsorbed solvent is removed, and any extrinsic porosity between molecules is commonly lost during desolvation.^[17]^ Various strategies have been developed^[18]^ to retain porous structures in molecular crystals after activation, such as introducing hydrogen bonding interactions between building blocks or a second molecule that matches the size and shape of the unstable voids.^[17a]^ Surprisingly, we find here that **TAMC** can retain essentially its original packing after losing 11.2 wt.% from the solvated structure when the EA is removed, without any obvious strong and directional intermolecular interactions between **TAMC** molecules, such as hydrogen bonding.

**Figure 4:**
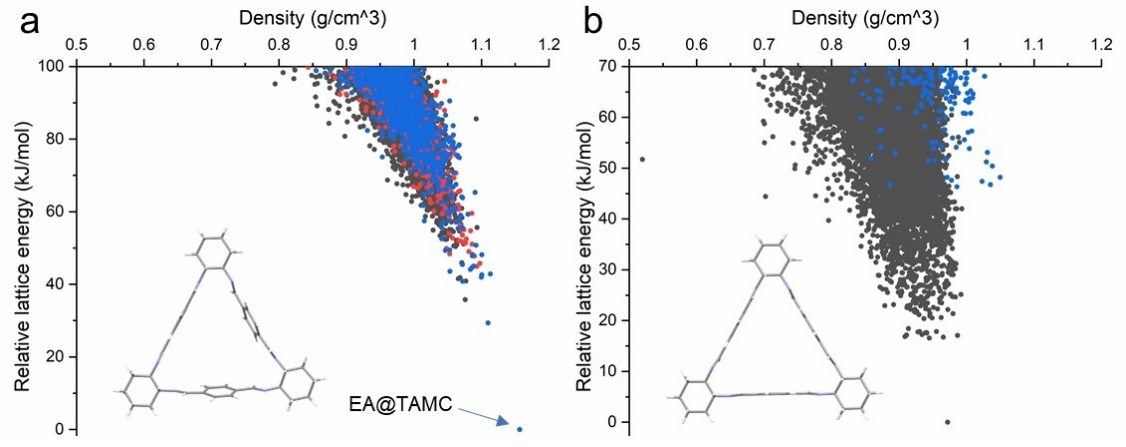
Crystal structure prediction (CSP) landscapes for a 1 : 1 composition of EA:**TAMC** in 6 common space groups (a) using the conformation from the experimentally determined EA@**TAMC** crystal structure; (b) using the calculated gas phase minimum conformer from a conformational search (Figure S25). Each point here corresponds to a predicted crystal structure that is a local minimum on the lattice energy surface. The color‐coding of structures is given by the positioning of the EA molecule relative to the **TAMC** cavity. Red dots represent structures in which the methyl end of EA sits inside **TAMC**, blue dots are structures where the ethyl end of EA is inside the **TAMC** cavity and black dots are structures where EA is outside **TAMC**. Note that there are no red dots in (b).

To probe this stability, the host‐guest chemistry of **TAMC** with EA was investigated by performing crystal structure prediction (CSP). To investigate how the macrocycle's flexibility contributes to the adsorption process, we calculated the landscapes of the possible 1 : 1 EA:**TAMC** co‐crystal structures available to both the conformation that was extracted from the experiment for EA@**TAMC** crystal structure (Figure 4a) as well as the lowest energy gas phase conformer calculated in a conformational search (Figure 4b). The structural difference between these two conformers is shown in Figure S26. The predicted crystal structures were classified according to the positioning of EA relative to the **TAMC** cavity: structures where the EA is outside of the **TAMC** cavity are shown by black points in Figure 4, those with the methyl group or ethyl group inside the cavity are represented by red points and blue points, respectively. In EA@**TAMC**, the ethyl group of EA sits inside the **TAMC** cavity, with its methyl group forming three C−H⋅⋅⋅π hydrogen bonds with the three phenyl rings of **TAMC**. An additional hydrogen bond is formed between the carbonyl of EA and another **TAMC**.

Although the molecular geometry in the observed EA@**TAMC** crystal structure and the gas phase optimized conformation differ only by small rotations of the phenyl rings, this modest difference has a large impact on the **TAMC**’s ability to adsorb EA. The observed behavior is reproduced in the crystal structure landscape derived from the observed molecular conformation: most low‐energy structures are densely packed with either the methyl or ethyl group of the EA molecule located inside the **TAMC** cavity. The lowest energy predicted structures have the ethyl group of EA located in this cavity (Figure 4a). The lowest energy structure accurately reproduces the experimental crystal structure, EA@**TAMC** (Figure 4a). By contrast, EA remains outside the **TAMC** cavity in the majority of the predicted crystal structures when the gas phase conformer is used (Figure 4b); none has the methyl end inside the cavity and only high‐energy predicted structures (more than 35 kJ/mol above the global minimum) have the ethyl end inside the **TAMC** cavity. To further verify this, a more complete CSP search in an expanded set of space groups was performed with the gas phase molecular geometry (Figure S27), confirming that EA does not fit within the cavity without distortion of the macrocycle.

These results demonstrate the importance of flexibility in guest adsorption.^[11a]^ The rigid backbone of **TAMC** retains the inherent pore in the structure after activation, while the subtle flexibility associated with rotation of the phenyl rings allows the macrocycle cavity to adapt for the ethyl group in EA, thus explaining both the adaptability and the stability of EA@**TAMC** crystal. To verify the energy ranking, all structures from the lowest 20 kJ/mol from both landscapes were re‐optimized with solid state density functional theory (DFT) with no geometry constraints (Supporting Information Section 8.3 for details). These more accurate calculations find the observed EA@**TAMC** structure 22 kJ/mol lower in energy than all other predicted alternatives (Figure S28). This result further supports that the experimental EA@**TAMC** structure is the global minimal packing structure.

We also performed CSP calculations on guest‐free **TAMC** using the gas phase conformer, followed by DFT re‐optimization of the predicted structures. The observed experimental structure was located among the predicted structures, 50 kJ/mol above the lowest energy predicted structure. This energy gap is comparable to other desolvated, porous molecular crystals that can be accessed experimentally.^[19]^ It seems that the EA interactions with the host **TAMC** structure stabilize the observed EA@**TAMC** phase during crystallization, and that this structure occupies a sufficiently deep energy basin such that *α*‐**TAMC** does not rearrange after desolvation. At least on the experimental timescales investigated here, even though we estimate that there are packings available to this macrocycle that are around 50 kJ/mol more stable. Hence, we only observe a subtle structural difference between the packing of **TAMC** molecules in EA@**TAMC** and *α*‐**TAMC**. This has practical importance because while other **TAMC** polymorphs can transform to EA@**TAMC**, and hence show EA selectivity (see discussion above), the kinetics are significantly slower than for *α*‐**TAMC**. As such, the structural template of EA in *α*‐**TAMC** improves the prospects for practical separation processes.

The electrostatic surface potential (ESP) of EA and EtOH with the most positive ESP (V_s,max_) and most negative ESP (V_s,min_) are presented in Figure S29. To give a deep insight into the intermolecular interaction model, the noncovalent index (NCI)^[20]^ theory was adopted to provide a more global description of the interaction between hosts and guests. As shown in Figure S30, there is a strong C−H⋅⋅⋅O hydrogen bonding interaction between the carbonyl group on EA and cyclohexane ring on the second **TAMC**. One **TAMC** captured the methyl group of one EA molecule with each benzene ring forming a C−H⋅⋅⋅π interaction with one methyl group C−H, leading to this methyl end being captured in the center of the **TAMC** cavity. With EA extended in the space, another end of EA interacts with the benzene ring on another **TAMC**, as shown by the green iso‐surface on the NCI plot. The interaction energy between **TAMC** and EtOH (‐0.62 eV) is weaker than the interaction energy between **TAMC** and EA (−0.91 eV) according to these first principles calculations. This can be attributed to both the weak V_s,max_ of hydrogen bond sites and loss of C−H⋅⋅⋅π interaction on the hydroxyl end of EtOH.

## Conclusion

In summary, we have found that a known macrocycle molecule, **TAMC**, can selectively adsorb EA, forming a stable EA@**TAMC** complex in the solid state, as confirmed by crystal structure prediction calculations. The flexible structure of the guest‐free *α*‐**TAMC** solid allows the formation of perfect complementary voids for ethyl acetate, which leads to selective adsorption of EA. As a result of this inherently high selectivity, *α*‐**TAMC** shows great promise for the dynamic separation of EA from EA‐EtOH mixtures, as confirmed by breakthrough experiments. **TAMC** is easily synthesized, shows good reliability after multiple adsorption cycles, and holds strong promise for practical separation or detection applications in the future. More generally, the concept of solvent‐templated molecular crystals, stabilized because they occupy deep energy basins on their structure landscapes, might be extended to other molecular separations in the future.

## Conflict of interest

The authors declare no conflict of interest.

## Supporting information

As a service to our authors and readers, this journal provides supporting information supplied by the authors. Such materials are peer reviewed and may be re‐organized for online delivery, but are not copy‐edited or typeset. Technical support issues arising from supporting information (other than missing files) should be addressed to the authors.

Supporting Information
