# Supplementary material for: Inherent Ethyl Acetate Selectivity in a Trianglimine Molecular Solid
Source: Chemistry. 2021 May 27;27(41):10589–94. doi: 10.1002/chem.202101510 (PMC8362070; doi:10.1002/chem.202101510)
Supplement: Supplementary file 1 — Supporting Information [file CHEM-27-10589-s001.pdf]

Chemistry–A European Journal

Supporting Information

Inherent Ethyl Acetate Selectivity in a Trianglimine Molecular Solid

Donglin He[+], Chengxi Zhao[+], Linjiang Chen, Marc A. Little, Samantha Y. Chong, Rob Clowes, Katherine McKie, Mark G. Roper, Graeme M. Day,* Ming Liu,* and Andrew I. Cooper*

Contents

1. Supplemental Experimental Procedures 2

1.1. Materials 2

1.2. Synthesis of **TAMC** 2

1.3. Activation of α-**TAMC** 2

1.4. Crystallisation of **TAMC** solvates 2

1.5. Methods 3

1.5.1 Solution NMR: 3

1.5.2 Thermogravimetric analysis: 3

1.5.3 Powder X-ray diffraction (PXRD): 3

1.5.4 Single crystal X-ray diffraction (SC-XRD): 3

1.5.5 Vapor phase isotherm adsorption (gravimetric apparatus): 4

1.5.6 Gas sorption: 4

2. Crystallography report 5

3. Characterization of **TAMC** and EA@**TAMC** 6

4. Vapor-phase adsorption measurements 13

4.1. Time-dependent **TAMC** solid−vapor sorption for EA/EtOH vapor 13

4.2. Selective uptake from an EA-EtOH mixture vapor in **TAMC** based on NMR 16

4.3. Selective uptake from an EA-EtOH azeotropic vapor mixture in **TAMC**, as measured by NMR 17

4.4. Single-component EA-EtOH adsorption experiment in **TAMC** as measured by IGA 18

4.5. Selective EA adsorption from a low concentration of a EA-EtOH mixture in **TAMC** 19

5. Gas sorption result of **TAMC** 21

6. Polymorph screening for **TAMC** 23

6.1. PXRD characterizations of **TAMC** polymorphs 23

6.2 Selective uptake from an EA-EtOH mixture in α-**TAMC** and **TAMC** polymorphs 29

7. Breakthrough experiments 31

8. Computational details: 32

8.1 Conformational search 32

8.2 CSP method 33

8.3 Periodic DFT re-optimization 34

8.4 Substructure search 34

8.5 Ideal adsorbed solution theory (IAST) 34

8.6 ESP and NCI analysis 35

8.7 Interaction energy calculation 35

References 41

1. Supplemental Experimental Procedures

1.1. Materials

(1*R*,2*R*)-Cyclohexane-1,2-diamine was purchased from Manchester Organics, UK. All other chemicals were purchased from *Sigma-Aldrich* and used as received.

1.2. Synthesis of TAMC

TAMC was synthesized as described previously.[1] Terephthaldehyde (1.34 g, 10 mmol) in dichloromethane (8.3 ml) was added to a solution of (1*R*,2*R*)-diaminocyclohexane (1.14 g, 10 mmol) in dichloromethane (5 ml). The mixture was stirred at room temperature for 3 h. The solvent was evaporated under vacuum, to afford the crude compound as a white powder. **TAMC** was recrystallized from ethyl acetate (EA) to afford EA@**TAMC** as colorless needles.

1.3. Activation of α-TAMC

To generate activated *α*-**TAMC**, crystals of EA@**TAMC** were initially dried under vacuum at room temperature for 5 h, and then fully activated after heating EA@**TAMC** at 70 °C under vacuum for 12 h. The single crystal of guest-free, α-**TAMC**, was obtained by activating crystals of EA@**TAMC** using this procedure.

1.4. Crystallisation of TAMC solvates

1 mL of the various organic solvents listed in Table S1 were added to 10 mg **TAMC** in separate glass sample vials. The solvents were then allowed to evaporate from the vials at room temperature, which took between 2–15 days, and the crystals were collected.

Table S1. Solvents for crystallization

Solvent	Boiling Point (°C)	Solvent	Boiling Point (°C)
Acetone	56.3	Methanol	64.7
Acetonitrile	81.6	*iso*-Propanol	82.3
Chloroform	61.2	*n*-Propanol	97.2
Cyclohexane	80.7	Tetrahydrofuran	66.0
Dichloromethane	39.8	Toluene	110.6
1,4-Dioxane	101.0	Triethyl orthoformate	148.2
1,4-Difluorobenzene	88-89	Water	100.0
Ethanol	78.3	*o*-Xylene	144.4
Heptane	98.4	*m*-Xylene	139.1
n-Hexane	68.7	*p*-Xylene	138.5

1.5. Methods

1.5.1 Solution NMR: Solution ^{1}H NMR spectra were recorded at 400.13 MHz using a Bruker Avance 400 NMR spectrometer.

1.5.2 Thermogravimetric analysis: TGA analysis was carried out using a Q5000IR analyzer (TA instruments) with an automated vertical overhead thermobalance. The samples were heated at the rate of 5 °C /min using dry N_2 as the protective gas.

1.5.3 Powder X-ray diffraction (PXRD): For screening, PXRD patterns were collected in transmission mode on samples held on thin Mylar film in aluminum well plates on a Panalytical Empyrean diffractometer, equipped with a high throughput screening XYZ stage, X-ray focusing mirror, and PIXcel detector, using Cu-Kα (λ = 1.541 Å) radiation. Unless stated, PXRD patterns were recorded at room temperature. High resolution variable temperature PXRD for EA@**TAMC** was collected using the I11 beamline at Diamond Light Source (λ = 0.82446 Å), using the Mythen II position sensitive detector. The finely ground sample was loaded into a 0.5 mm diameter borosilicate glass capillary and exposed to ethyl acetate vapor for 48 hours at room temperature. A capillary spinner was used to improve powder averaging during data acquisition. The temperature was controlled using an Oxford Cryosystems Cryostream Plus. The sample was cooled to 223 K to record a reference profile of the solvated structure, then heated from 273 – 393 K, with data collections at 5 K steps. The sample temperature was maintained above 363 K for approximately 1.5 hours to effect desolvation. The sample was then cooled to 295 K to acquire the final diffraction profile. Indexing and Le Bail refinements were performed using TOPAS-Academic.[2]

1.5.4 Single crystal X-ray diffraction (SC-XRD): SC-XRD data sets were measured on a Rigaku MicroMax-007 HF rotating anode diffractometer (Mo-Kα radiation, λ = 0.71073 Å, Kappa 4-circle goniometer, Rigaku Saturn724+ detector); or at beamline I19, Diamond Light Source, Didcot, UK using silicon double crystal monochromated synchrotron radiation (λ = 0.6889 Å, Pilatus 2M detector). Absorption corrections, using the multi-scan method, were performed with the program SADABS.[3] For synchrotron X-ray data, collected at Diamond Light Source, data reduction and absorption corrections were performed with xia2.[4] Structures were solved with SHELXT,[5] and refined by full-matrix least-squares on $|F|^2$ by SHELXL,[6] interfaced through the program OLEX2.[7] H atom positions for the C-H groups were refined

using the riding model. During crystal activation, the crystals of EA@**TAMC** break up into small fragments. Consequently, the X-ray diffraction intensity for the activated α-**TAMC** crystal was very weak. Synchrotron X-ray data was essential for structure solution. However, due to the poor X-ray data quality, a 1.0 Å resolution limit was applied during refinement, the aromatic rings of **TAMC** were refined with constrained geometries (AFIX66 in SHELX), and the **TAMC** molecule was refined with a rigid-bond restraint. Supplementary CIFs, which include structure factors, are available free of charge from the Cambridge Crystallographic Data Centre (CCDC) for EA@**TAMC** (2049238) and α-**TAMC** (2049237).

1.5.5 Vapor phase isotherm adsorption (gravimetric apparatus): Vapor sorption was measured using an IGA-002 gravimetric sorption analyzer (*Hiden Isochema*, Warrington, UK) with a weight measurement resolution of 0.2 μg and long term stability of +/- 1 μg. Approximately 25 mg of **TAMC** was loaded in a gas permeable stainless steel mesh pan and degassed in situ at 343 K under high vacuum ($1x10^{-6}$ mbar) for a minimum of 4 hours until the sample mass was stable. Isotherms were measured from 0 to 0.9 P/P_0, with initial steps at 0.01 P/P_0 increments. The sample temperature was regulated using a water bath at 298 +/- 0.02 K throughout the isotherm measurements. The equilibration time for each isotherm point was determined automatically by the IGA-002 software, based on real-time analysis of the asymptotic sorption kinetic curve. The vapor pressure was held constant at each isotherm point with a typical regulation accuracy of +/- 0.02 mbar. Liquid solvents (ethyl acetate and ethanol) used to generate pure vapor were degassed fully in the IGA-002 by repeated evacuation and vapor expansion cycles prior to the measurements.

1.5.6 Gas sorption: Nitrogen adsorption and desorption isotherms of all **TAMC** were collected at 77 K using an ASAP2420 volumetric adsorption analyzer (Micrometrics Instrument Corporation). Carbon dioxide, methane, and hydrogen isotherms were collected up to a pressure of 1200 mbar on a Micromeritics ASAP2020 at 77 K for hydrogen, 273 and 298 K for carbon dioxide, 273 and 298 K for methane. All analogs were degassed at 70 °C for 15 hours under dynamic vacuum (10−5 bar) prior to analysis.

2. Crystallography report

Table S2. Experimental single crystal X-ray data for EA@**TAMC** and *α*-**TAMC.**

Molecule	EA@**TAMC**[a]	*α*-**TAMC**
Collection Temperature	100 K	100 K
Formula	$C_{46}H_{56}N_6O_2$	$C_{42}H_{48}N_6$
Mr [g mol^{-1}]	724.96	636.86
Crystal Size [mm]	0.152×0.313×0.533	0.026×0.022×0.019
Crystal System	Monoclinic	Monoclinic
Space Group	$P2_1$	$P2_1$
a [Å]	11.4675(10)	11.685(2)
b [Å]	10.2584(8)	9.759(2)
c [Å]	18.5370(16)	18.239(4)
α [°]	90	90
β [°]	105.090(2)	106.755(18)
γ [°]	90	90
V [Å^3]	2105.5(3)	1991.6(8)
Z	2	2
D_{calcd} [g cm^{-3}]	1.143	1.062
μ [mm^{-1}]	0.071	0.060
F(000)	780.0	684
2*θ* range [°]	1.89 – 30.033	2.295 – 24.999
Reflections collected	30809	13154
Independent reflections, R_{int}	9838, 0.0427	4048,0.1702
Obs. Data [$I > 2\sigma(I)$]	9221	2203
Data /restraints / parameters	9838/1/489	4048/379/397
Final R1 values ($I > 2\sigma(I)$)	0.0366	0.0687
Final R1 values (all data)	0.0394	0.1118
Final wR(F_2) values (all data)	0.0943	0.1665
Goodness-of-fit on F^2	1.051	0.913
Largest difference peak and hole [e.A^{-3}]	0.350/-0.173	0.248/-0.136
CCDC	2049238	2049237

[a] X-ray data for EA@**TAMC** is comparable to the reported X-ray crystal of XAGXUY reported in the Crystal Structure Database.[8]

3. Characterization of TAMC and EA@TAMC

EA@**TAMC** was recrystallized from EA as colorless needles. The resultant crystals were filtered from solvent and dried under a high vacuum at room temperature. They were then further characterized by ^{1}H NMR, TGA, and PXRD.

TAMC after vapor adsorption, an open 5 mL vial containing 5.5 mg of guest-free **TAMC** adsorbent was placed in a sealed 20 mL vial containing 0.5 mL of pure EA, pure EtOH, or a EA/EtOH (1/1, v/v) mixture, respectively. The resultant crystals were evaporated at room temperature for 30-45 min. After that, they were further characterized by ^{1}H NMR and PXRD.

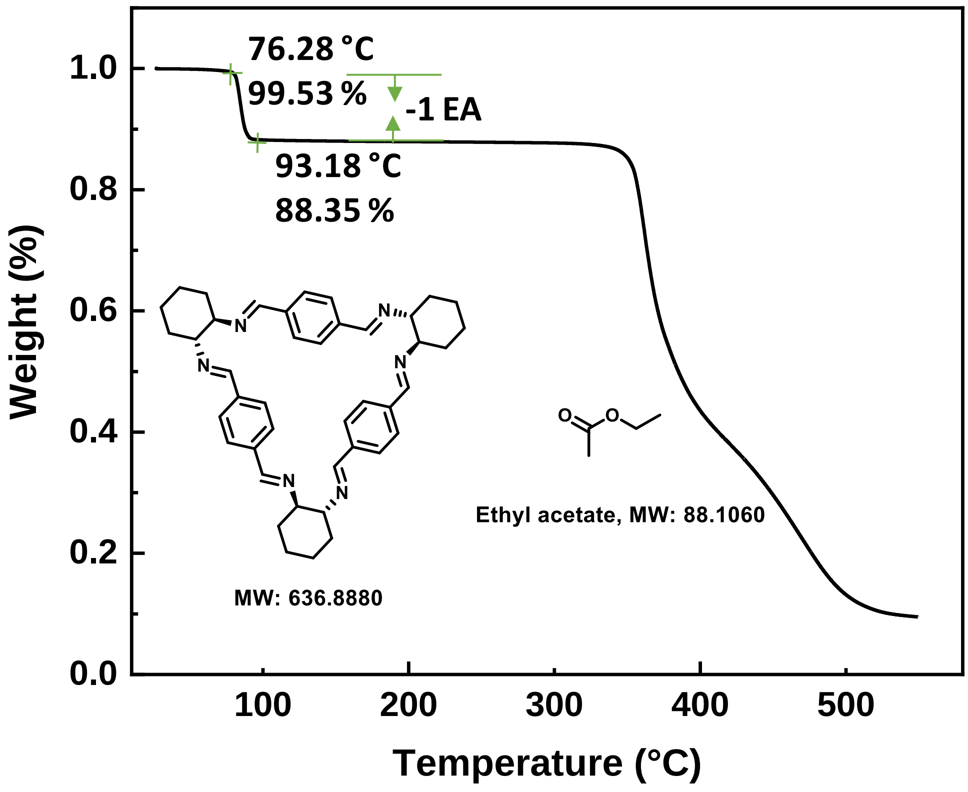

Figure S1. Thermogravimetric analysis of EA@**TAMC** crystal.

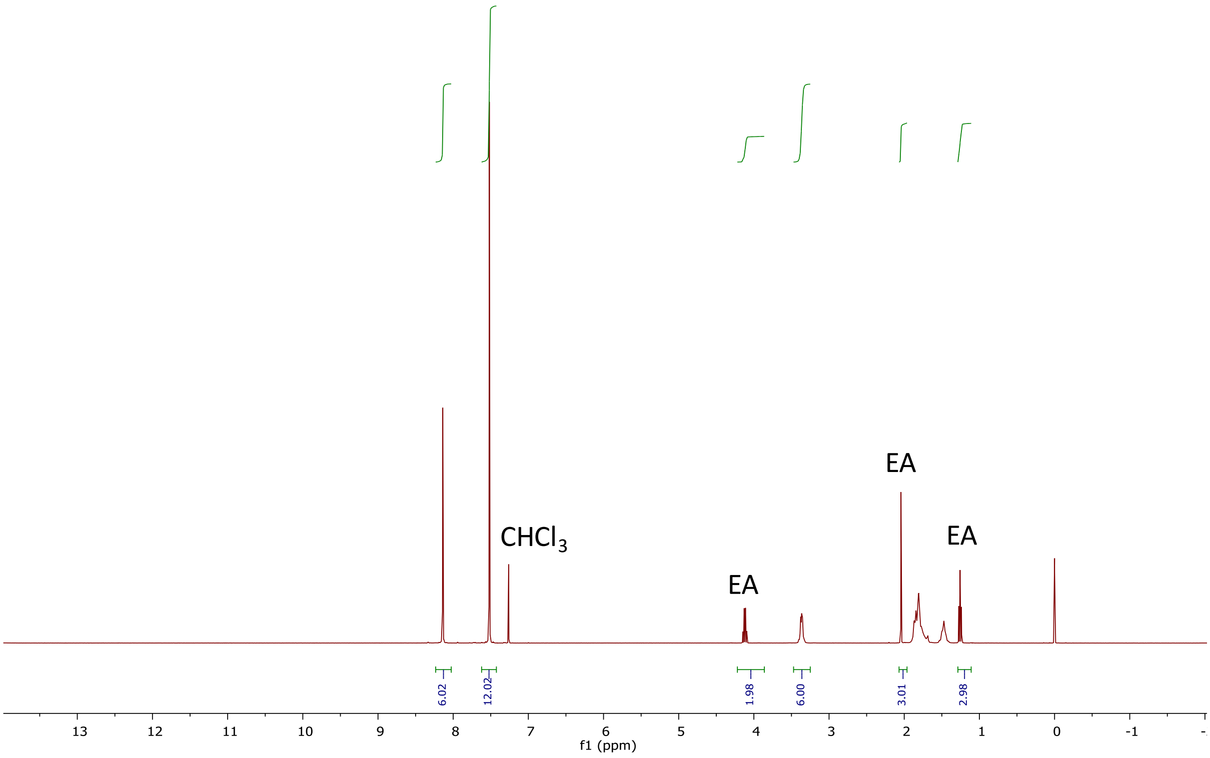

Figure S2. ^{1}H NMR spectrum (400 MHz, $CDCl_3$, 293 K) of EA@**TAMC**.

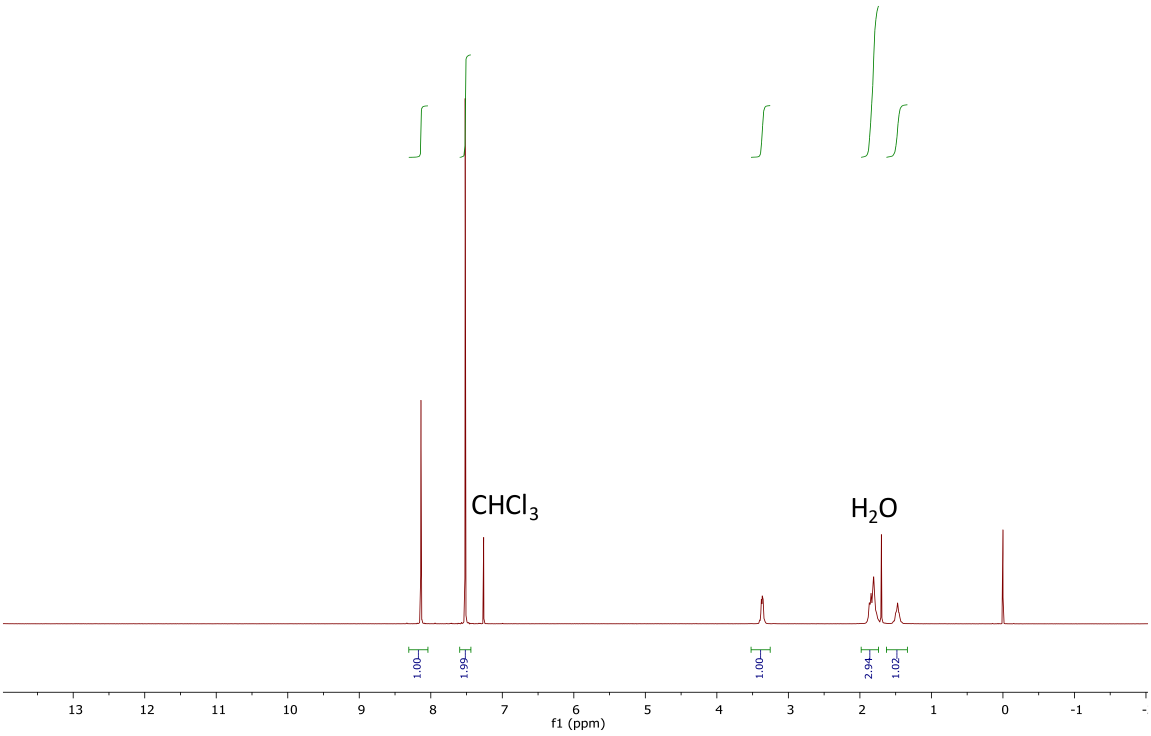

Figure S3. ^{1}H NMR spectrum (400 MHz, $CDCl_3$, 293 K) of guest-free *α*-**TAMC** desolvated at 70 °C.

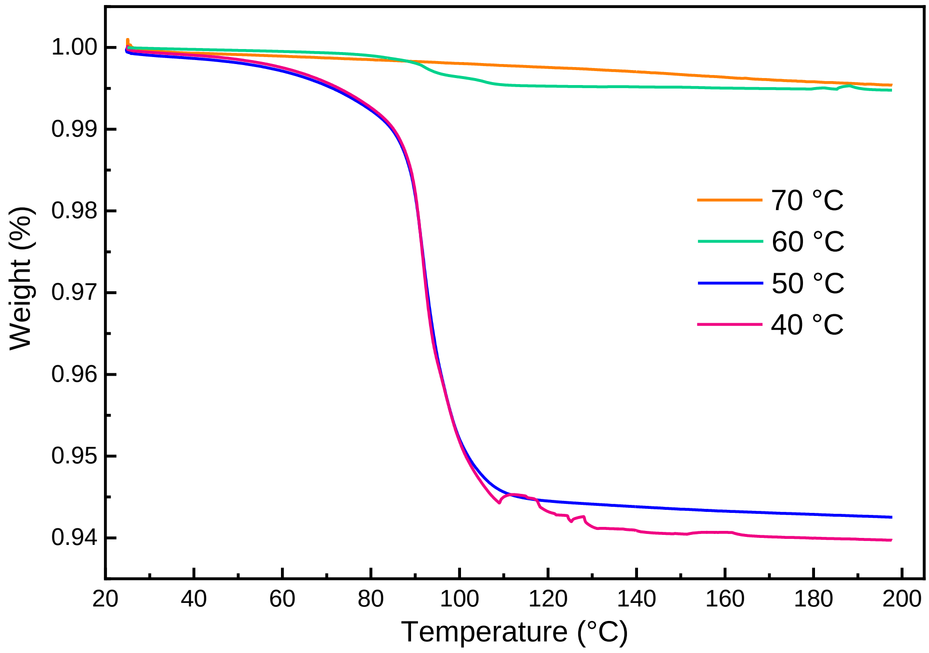

Figure S4. Thermogravimetric analysis of EA@**TAMC** samples that were heated at different temperatures (40–70 °C) under vacuum. A temperature of ≥70 °C was required to remove all of the EA.

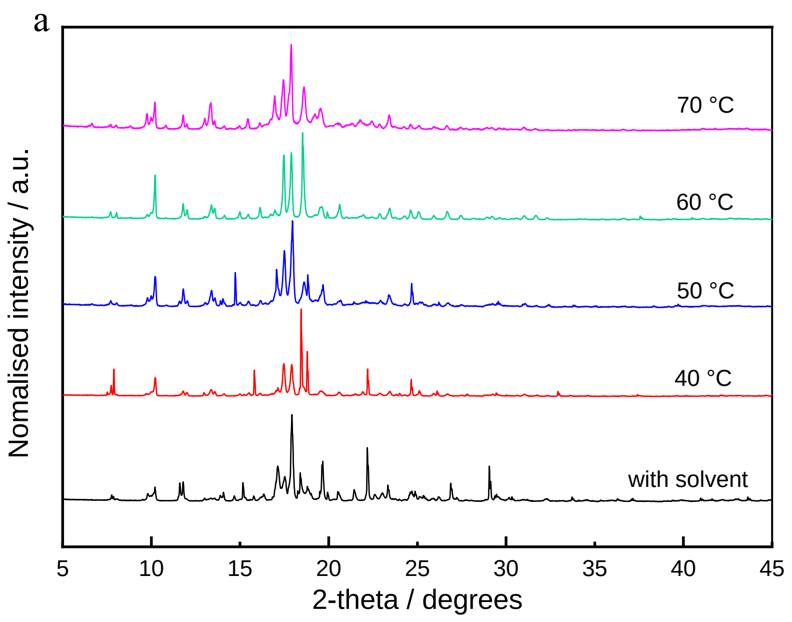

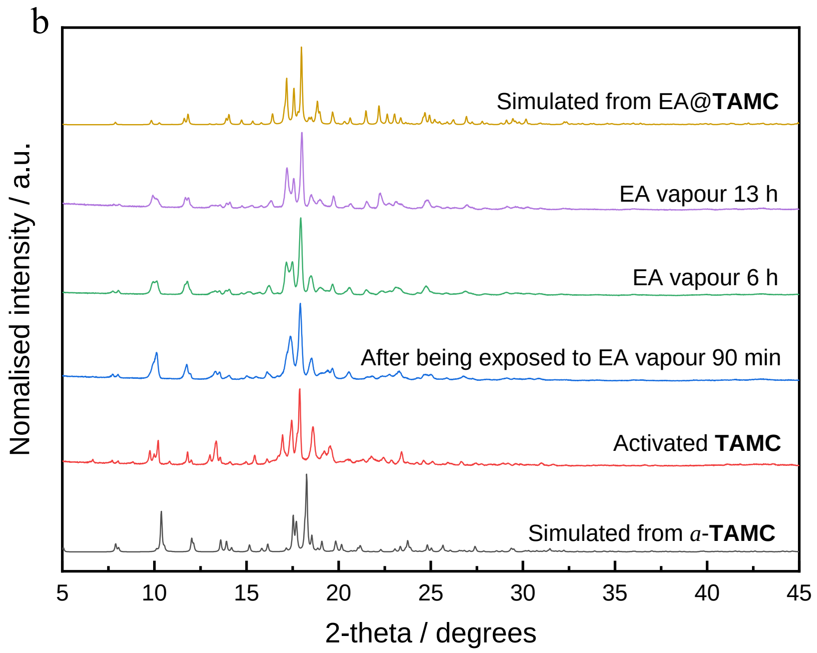

Figure S5. (a) PXRD patterns of EA@**TAMC** samples that were activated at different temperatures (40–70 °C) under vacuum. (b) Time-dependent PXRD patterns of activated **TAMC** solid after being exposed to EA vapor. After being exposed to EA vapor, the activated **TAMC** would gradually transform to EA@**TAMC** phase.

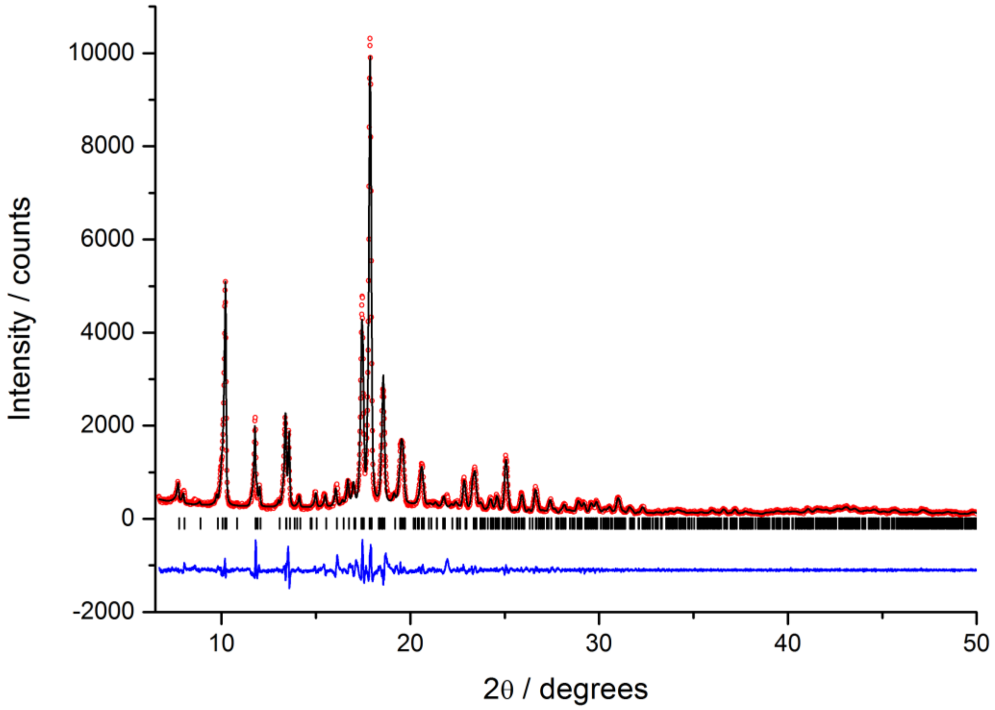

Figure S6. Final observed (red circles), calculated (black line) and difference PXRD profiles (Cu $K\alpha_{1,2}$) for Le Bail refinement of EA@**TAMC** activated at 80 °C under vacuum. The unit cell (a = 9.9394(7) Å, b = 11.7889(7) Å, c = 19.346(1) Å, α = 111.135(4)°, β = 88.750(4)°, γ = 89.749(4)°, V = 2113.7(2) Å^3, P1) indicates a structure similar to EA@**TAMC** and the single crystal, but with lowered symmetry and a volume consistent with two independent molecules in the asymmetric unit.

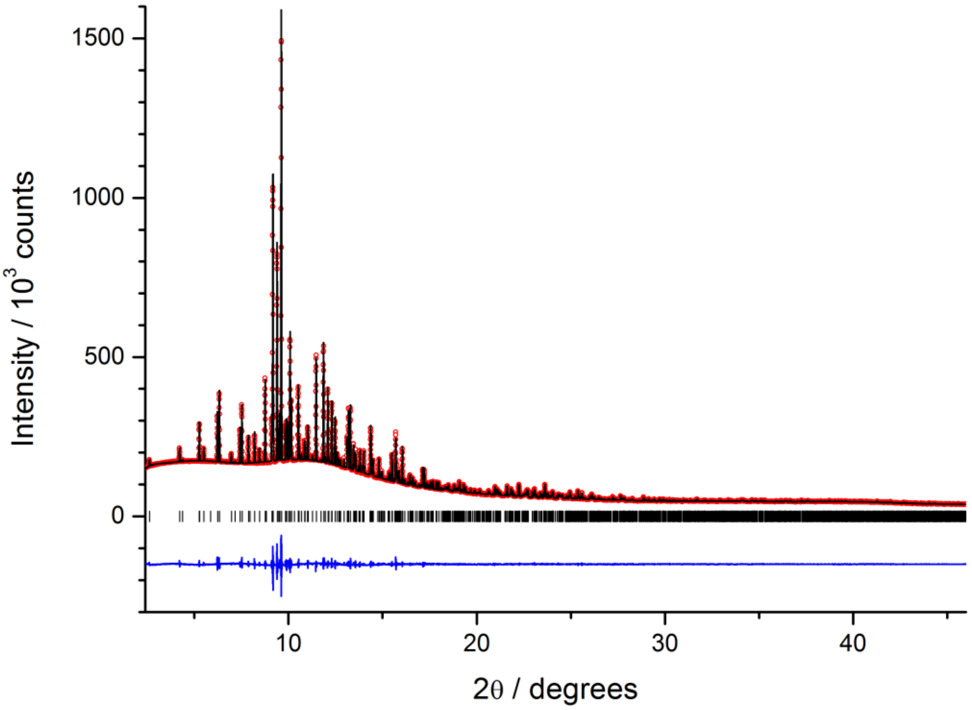

Figure S7. Final observed (red circles), calculated (black line) and difference PXRD profiles (λ = 0.82446 Å) for Le Bail refinement of EA@**TAMC** at 223 K (R_{wp} = 1.90%, R_p = 1.15%, χ^2 = 5.31), prior to *in situ* VT-PXRD measurement (Figure 1d). The refined unit cell (a = 11.5368(3) Å, b = 10.32149(4) Å, c = 18.57945(6) Å, $\alpha = \gamma$ = 90°, β = 104.5744(3)°, V = 2141.19(1) Å^3, $P2_1$) is consistent with the published structure for EA@**TAMC**.[9]

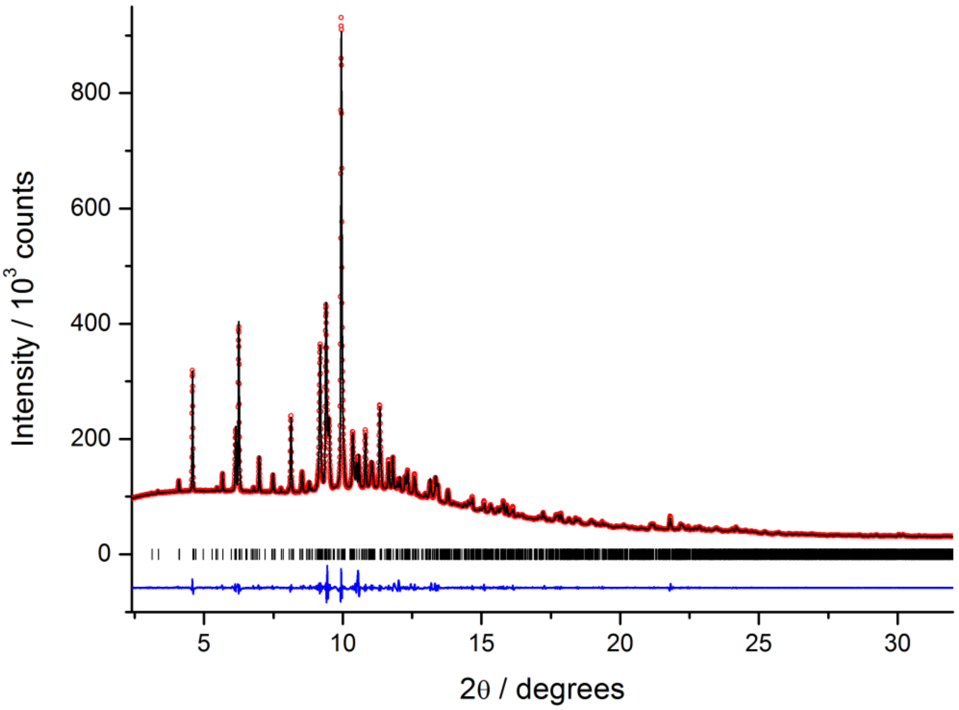

Figure S8. Final observed (red circles), calculated (black line) and difference PXRD profiles (λ = 0.82446 Å) for Le Bail refinement (R_{wp} = 1.90%, R_p = 1.15%, χ^2 = 5.31) for EA@**TAMC** after slowly heating *in situ* to 393 K at ambient pressure and subsequent cooling to 295 K to desolvate the powdered sample. *In situ* PXRD over the complete temperature range is shown in Figure 1d. The unit cell for the *in situ* heated bulk powder (a = 10.9148(1) Å, b = 16.1010(2) Å, c = 23.9228(3) Å, α = 98.959(1)°, β = 100.145(1)°, γ = 106.130(1)°, V = 3881.29(8) Å^3, P1) suggests a triclinic structure with a cell volume consistent with four molecules in the asymmetric unit, which may be related to polymorphs previously reported by Barbour *et al.*[10]

4. Vapor-phase adsorption measurements

4.1. Time-dependent TAMC solid−vapor sorption for EA/EtOH vapor

For each single-component EA/EtOH (1/1, v/v) adsorption experiment, an open 5 mL vial containing 5.5 mg of guest-free **TAMC** adsorbent was placed in a sealed 20 mL vial containing 1 mL of EA or EtOH. Uptake in the **TAMC** crystals was measured by completely dissolving the crystals in $CDCl_3$ and measuring the ratio of EA or EtOH to **TAMC** (mol/mol) by ^{1}H NMR, respectively.

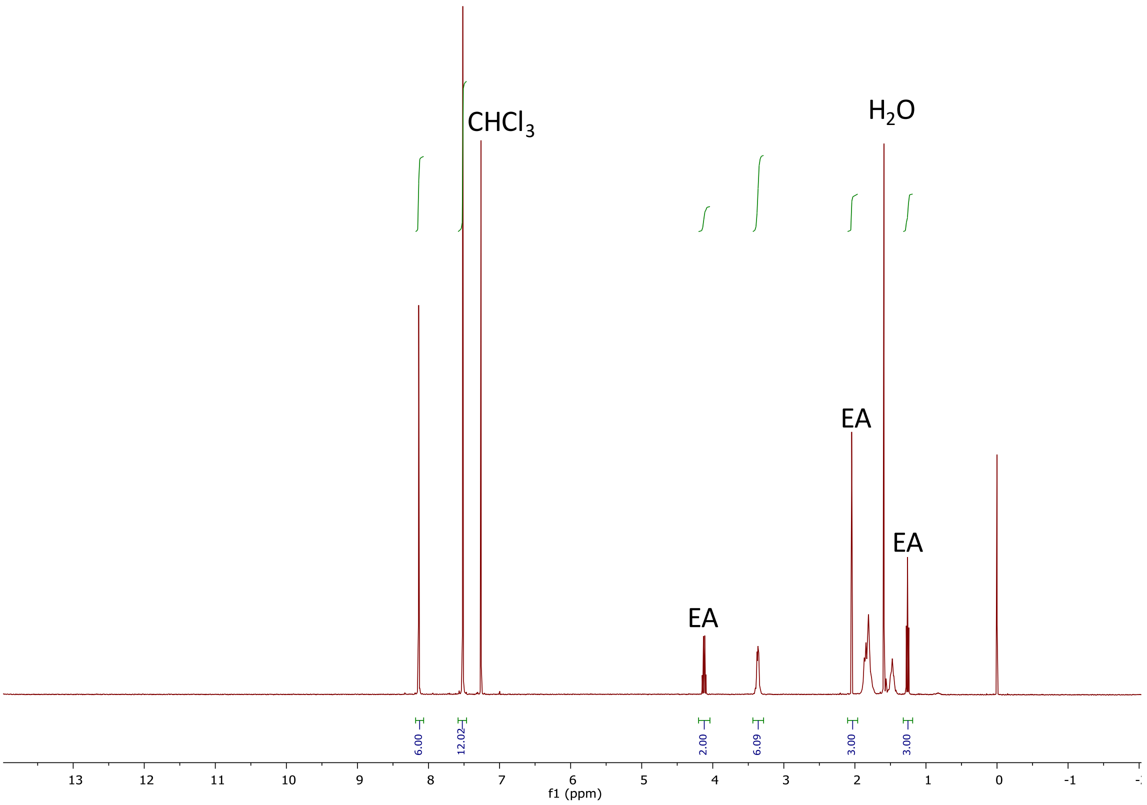

Figure S9. ^{1}H NMR spectrum (400 MHz, $CDCl_3$, 293 K) of **TAMC** after being exposed to EA vapor for 13 h.

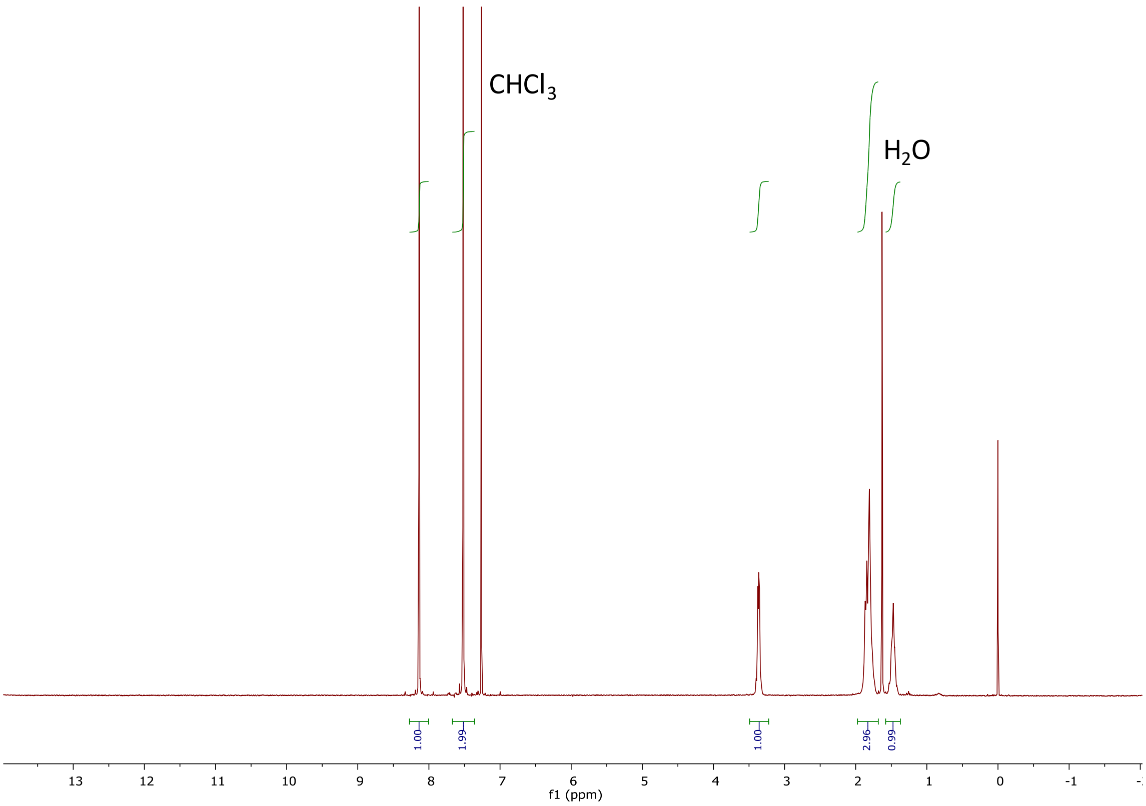

Figure S10. ^{1}H NMR spectrum (400 MHz, $CDCl_3$, 293 K) of **TAMC** after being exposed to EtOH vapor for 23 h.

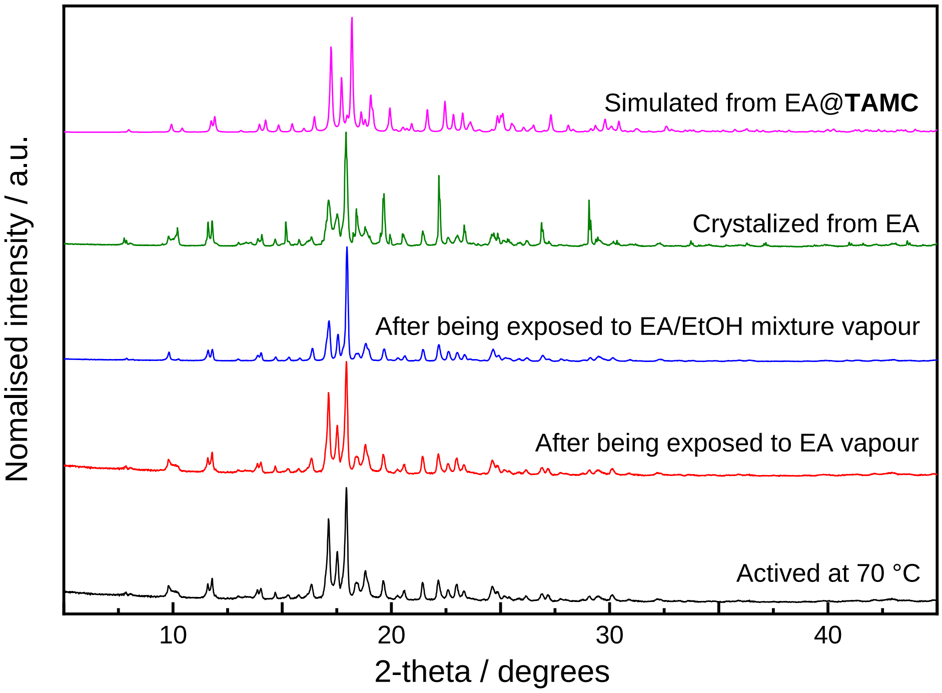

Figure S11. Powder X-ray diffraction patterns of **TAMC** at different conditions. From bottom to top: **TAMC** activated at 70 °C; after being exposed to EA vapor for 16 h; after being exposed to EA-EtOH vapor for 6 h; **TAMC** crystalized from EA (EA@**TAMC**); simulated from crystal structure EA@**TAMC**.

4.2. Selective uptake from an EA-EtOH mixture vapor in TAMC based on NMR

For each mixture vapor-phase experiment, an open 5 mL vial containing 5.5 mg of guest-free **TAMC** adsorbent was placed in a sealed 20 mL vial containing 0.5 mL of EA and EtOH, respectively. Uptake in the **TAMC** crystals was measured by completely dissolving the crystals in $CDCl_3$ and measuring the ratio of EA or EtOH to **TAMC** (mol/mol) by ^{1}H NMR, respectively.

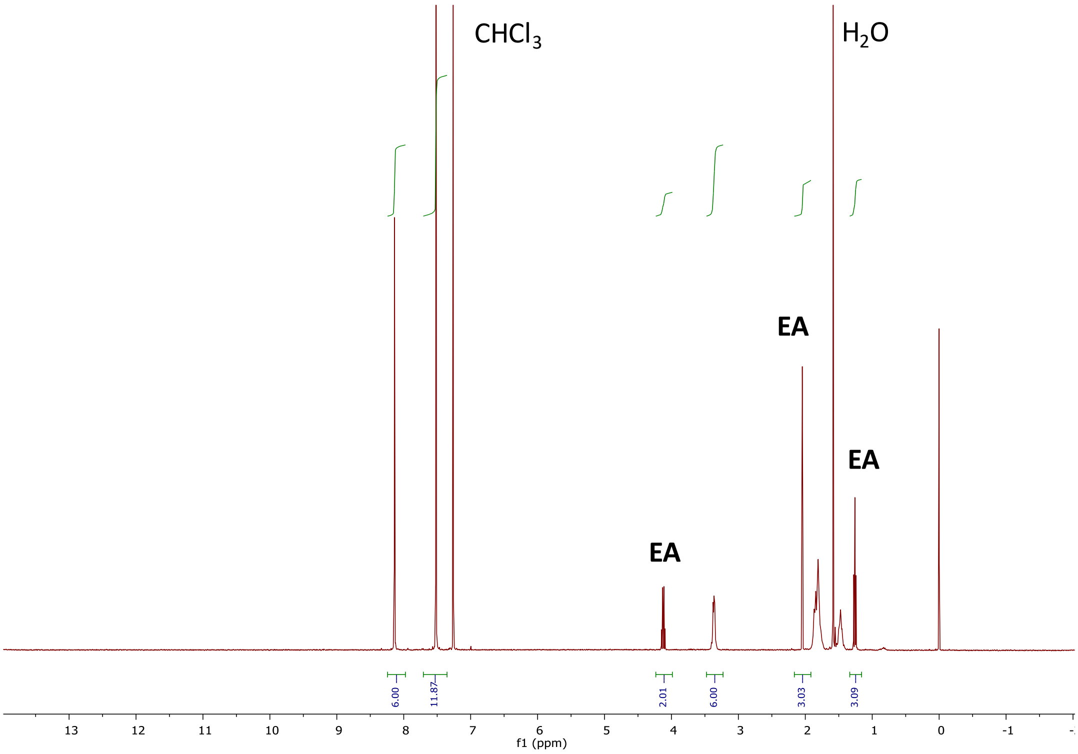

Figure S12. ^{1}H NMR spectrum (400 MHz, $CDCl_3$, 293 K) of **TAMC** after being exposed to EA-EtOH vapor mixture for 14 h.

4.3. Selective uptake from an EA-EtOH azeotropic vapor mixture in TAMC, as measured by NMR

For the azeotropic mixture vapor phase experiment, an atmospheric distillation apparatus was used to generate EA-EtOH azeotropic mixture vapor with 10 mL 30%/70% (w/w) EtOH-EA solvent. A cap of NMR tube containing 10 mg of guest-free **TAMC** adsorbent was placed below the arm of the distillation head by a long needle. Solvent uptake in the **TAMC** crystals was measured by completely dissolving the crystals in $CDCl_3$ and measuring the ratio of EA or EtOH to **TAMC** (mol/mol) by ^{1}H NMR, respectively.

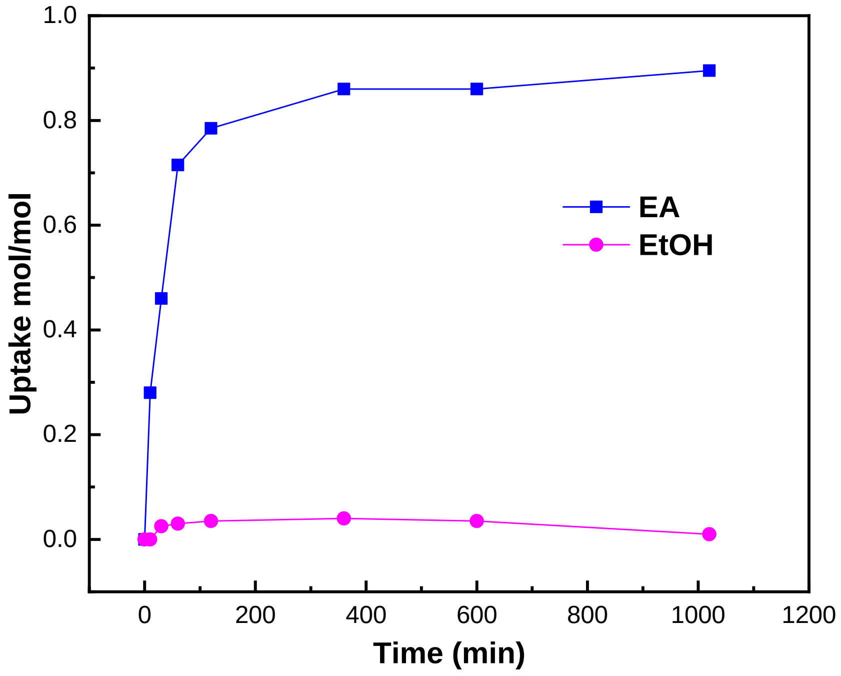

Figure S13. Time-dependent **TAMC** solid vapor sorption plot for EA/EtOH azeotropic mixture vapor. The macrocycle adsorbs EA but effectively rejects EtOH.

4.4. Single-component EA-EtOH adsorption experiment in TAMC as measured by IGA

The single-component EtOH and EA vapor isotherms for *α*-**TAMC** were measured by gravimetric sorption apparatus (IGA-002, Hiden Isochma) at 25 °C based on the change in sample mass as a function of pressure.

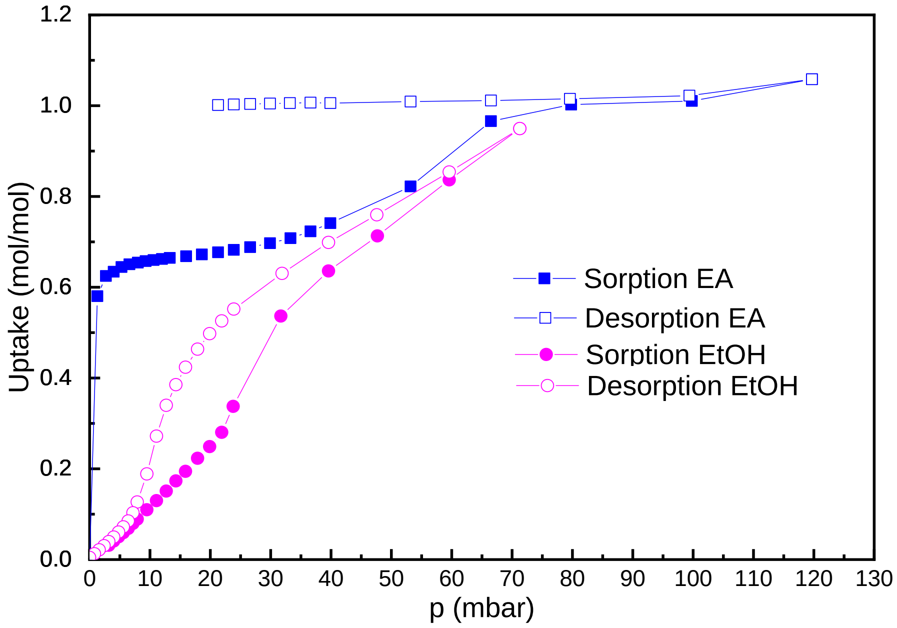

Figure S14. Single-component vapor sorption- desorption isotherms for *α*-**TAMC**.

4.5. Selective EA adsorption from a low concentration of a EA-EtOH mixture in TAMC

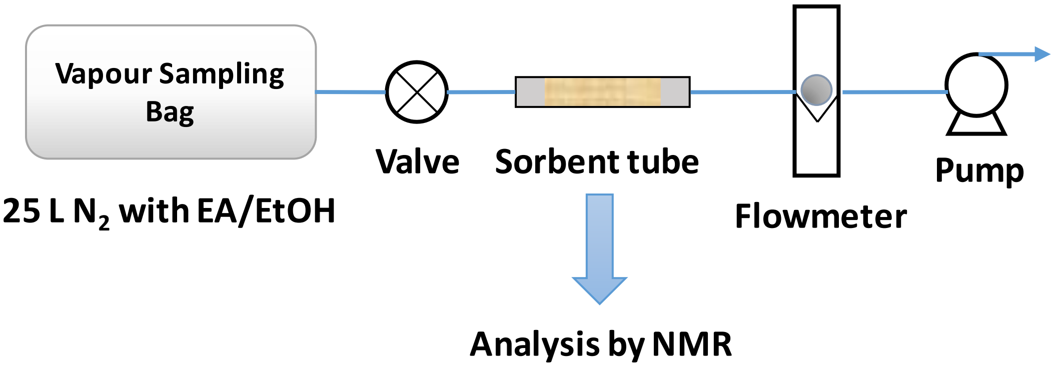

Figure S15. Schematic representation of the method to measure the selective uptake from a low concentration of EA-EtOH mixture in **TAMC**.

As shown in Figure. S15, activated *α*-**TAMC** solid (30 mg) was packed into empty thermal desorption tubes (I.D. × O.D. × L 4 mm × 6 mm × 4.5 in. made by *Dynatherm*) and plugged with a small amount of quartz wool. This was attached to a 25 L *Tedlar* bag containing the required EtOH-EA vapor mixture by using a short piece of silicone tubing. The EtOH-EA vapor mixture was generated by injecting liquid mixture (8 to 25 µL) with ratio of 50:50 v/v into the *Tedlar* bag. The pump with a flowmeter was attached to the other end of the tube, again using silicone tubing. The feed flow rate is 0.2 L/min. Uptake in the **TAMC** crystals was measured after 20 min exposure by completely dissolving the crystals in $CDCl_3$ and measuring the ratio of EA or EtOH to **TAMC** (mol/mol) by ^{1}H NMR, respectively. All the adsorption processes were carried out at room temperature.

Table. S3. The selective uptake from a low concentration of EA-EtOH mixture in **TAMC**

Maximum vapor concentration in Tedlar bag		Uptake of vapor	
EA (ppm)	EtOH (ppm)	EA (mol/mol)	EtOH (mol/mol)
124	210	0.015	0
254	425	0.025	0
508	850	0.04	0

5. Gas sorption result of TAMC

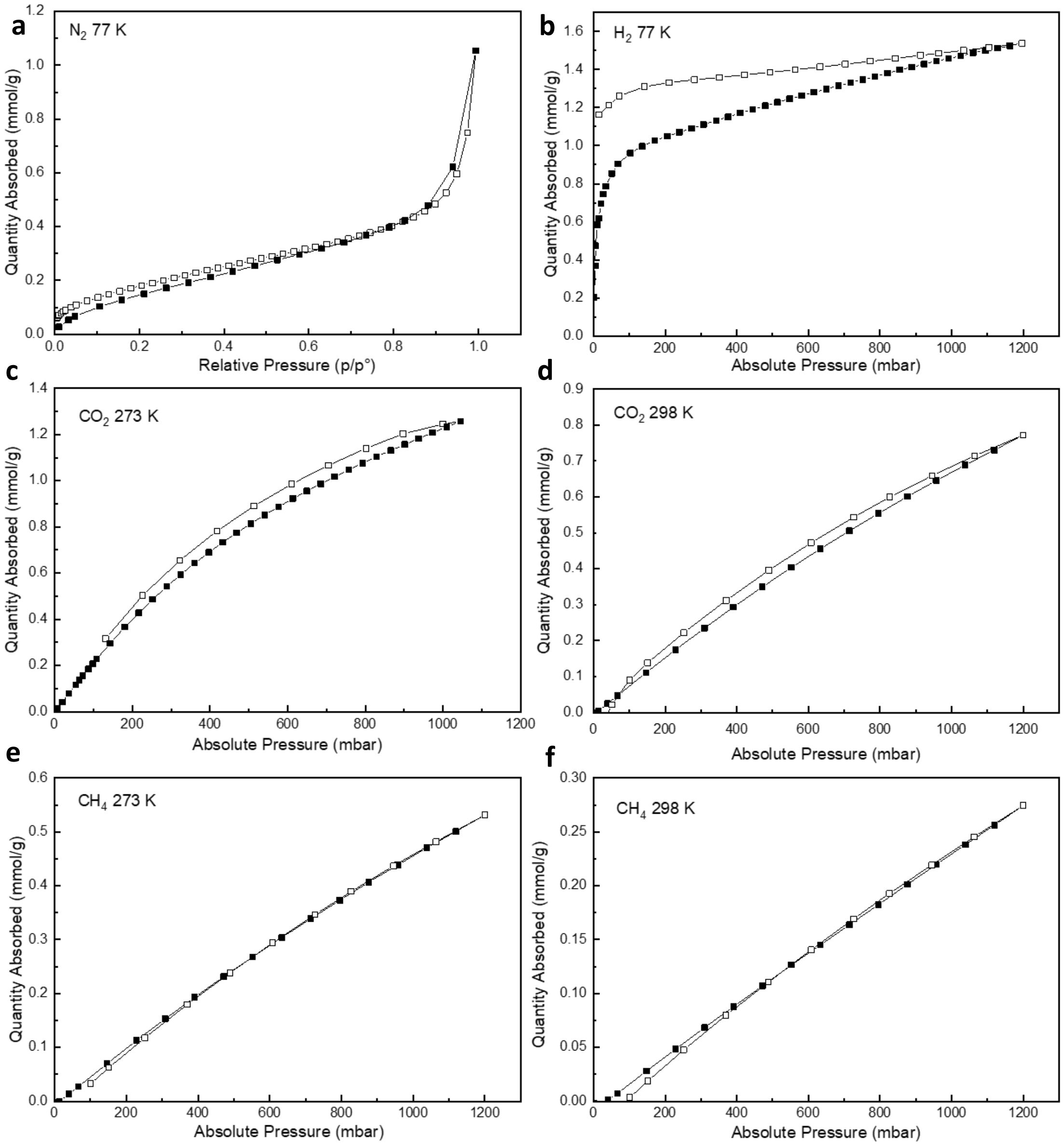

Figure S16. Gas adsorption / desorption isotherms for *α*-**TAMC**. (a) Nitrogen isotherms at 77 K. (b) Hydrogen at 77 K. (c) Carbon dioxide at 273 K. (d) Carbon dioxide at 298 K. (e) Methane at 273 K. (e) Methane at 298 K. Adsorption (filled symbols), desorption (hollow symbols).

As shown in Figure S16, *α*-**TAMC** has lower uptakes for CO_2 and CH_4 than for EA at comparable temperatures (298 K). Both EA and EtOH have much larger kinetic diameters than H_2, N_2, CO_2 and CH_4.[11] Therefore, we exclude molecular sieving as the cause for the EA/EtOH selectivity and believe instead that **TAMC** adsorbs more EA vapor because of the

stronger host-guest interaction between **TAMC** and EA. That is, the separation is driven by thermodynamics, not kinetics.

6. Polymorph screening for TAMC

6.1. PXRD characterizations of TAMC polymorphs

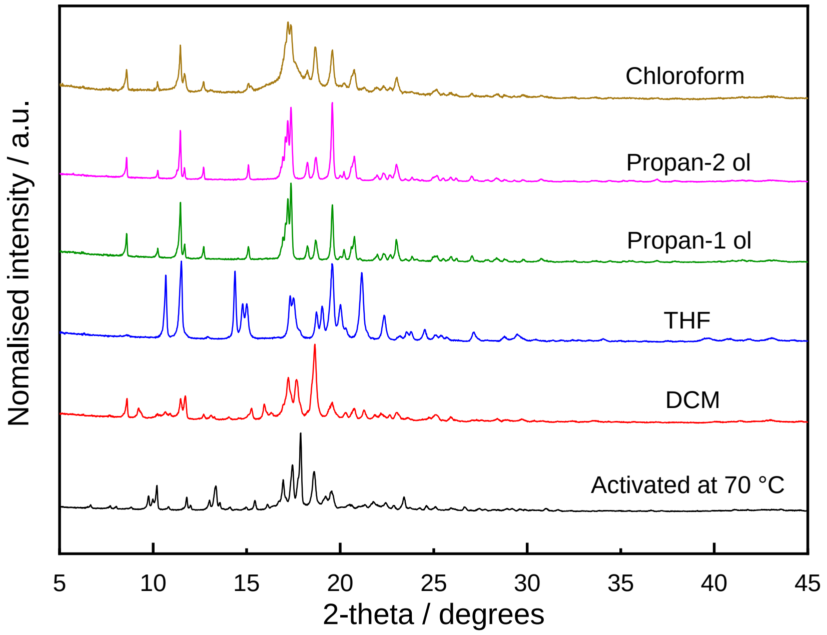

Figure S17. PXRD patterns of **TAMC** samples obtained from solvents that can dissolve **TAMC**.

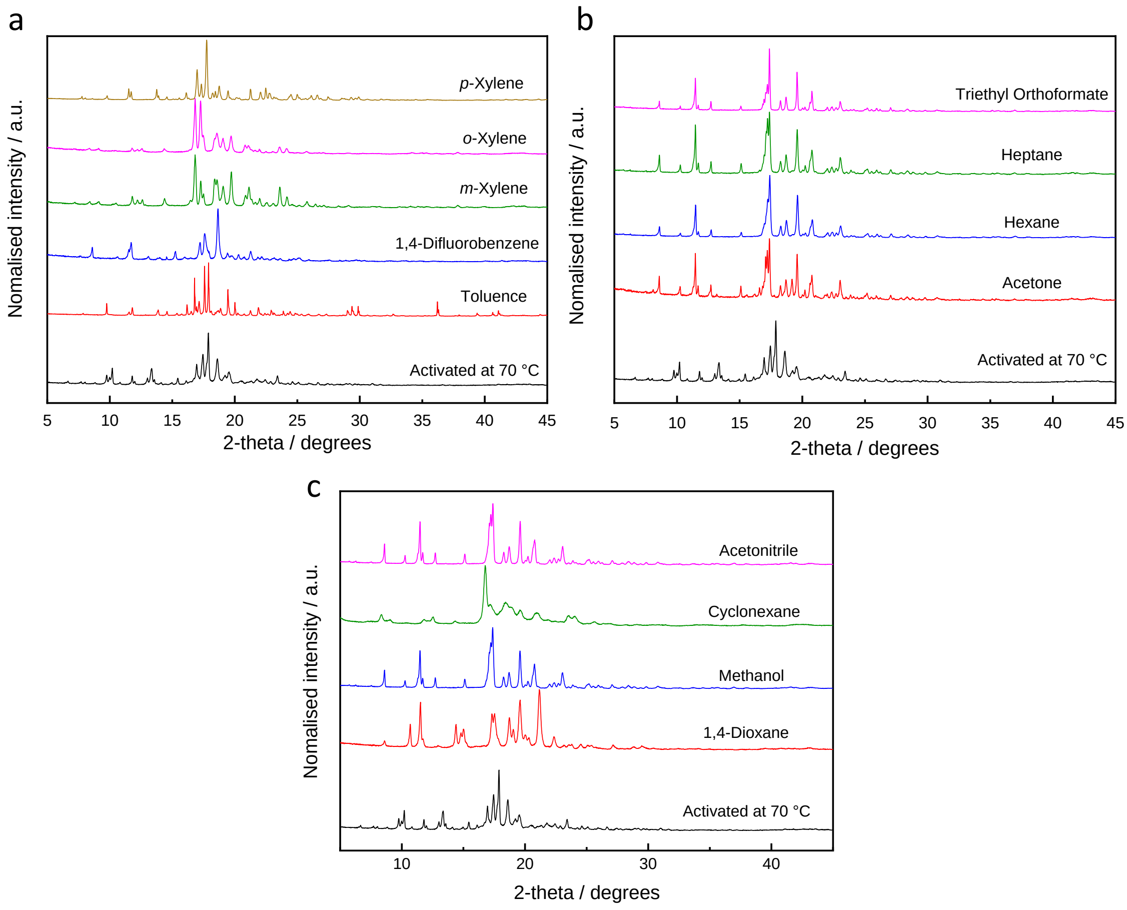

Figure S18. PXRD patterns of **TAMC** samples obtained from solvents that cannot dissolve **TAMC**.

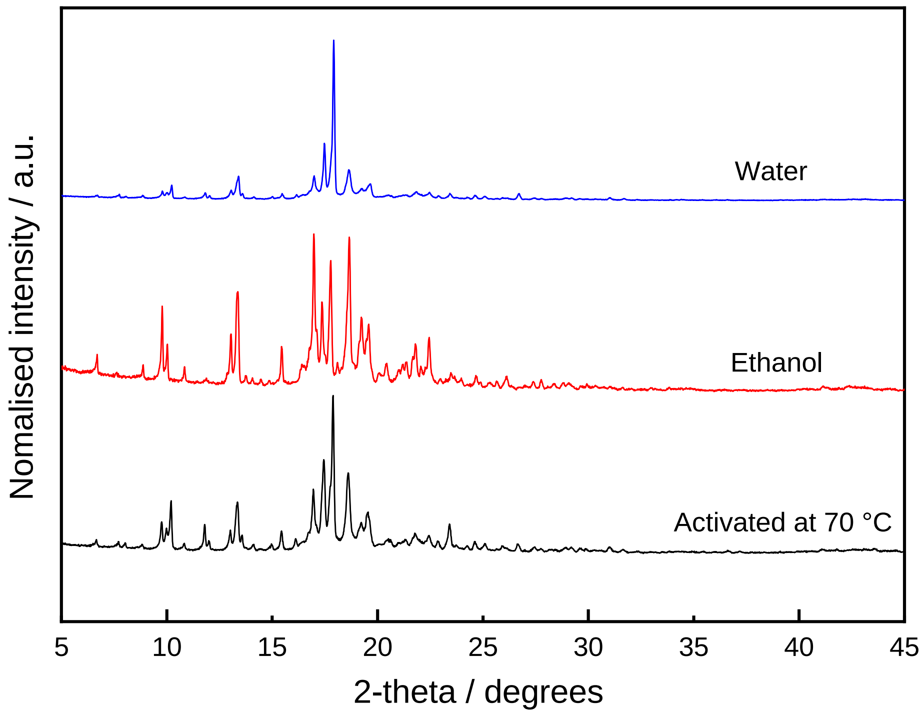

Figure S19. PXRD patterns of **TAMC** sample obtained from EtOH and H_2O

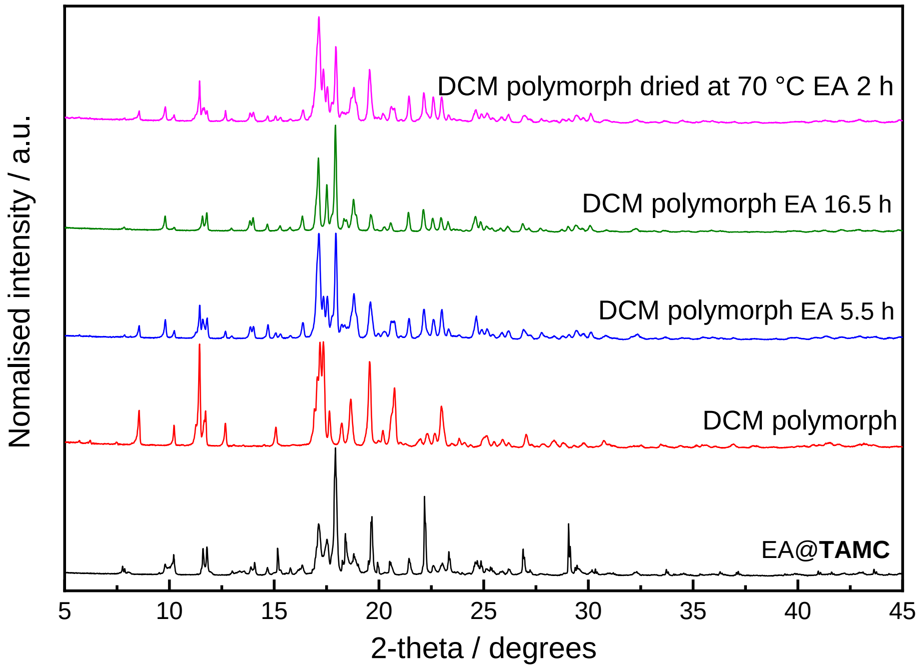

Figure S20. PXRD patterns of **TAMC** sample induced from **DCM.** From bottom to top: EA@**TAMC; DCM** polymorph**;** after adsorption of EA vapor for 5.5 h; after adsorption of EA vapor for 16.5 h; **DCM** polymorph dried (70 °C) after adsorption of EA vapor for 2 h**.**

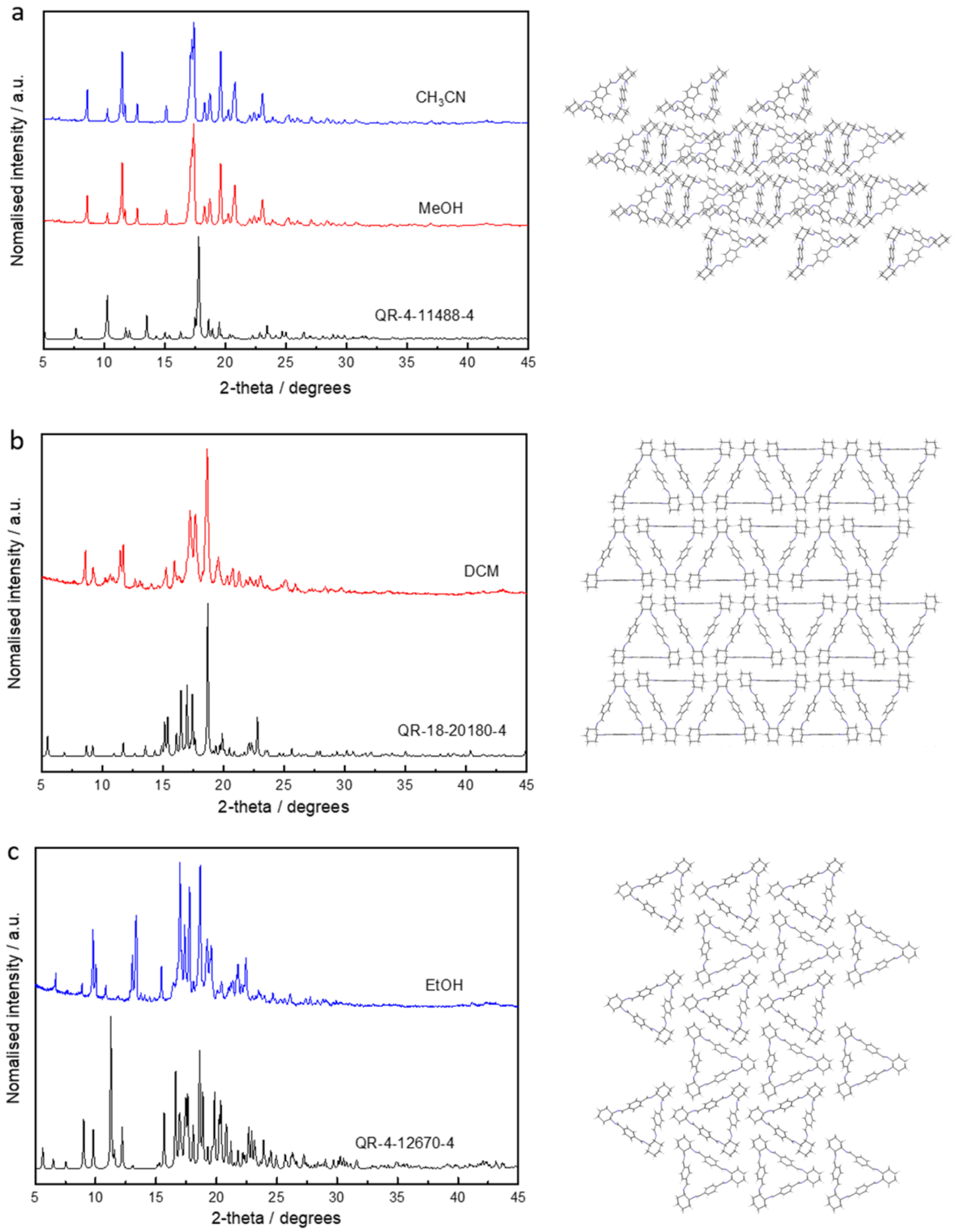

Figure S21. Potential matches between experimental **TAMC** polymorphs (from different solvents) with structures calculated from Crystal Structure Prediction. Color lines represent for experimental powder X-ray diffraction patterns of **TAMC** polymorphs and black lines represent the simulated crystal structures of **TAMC** from CSP calculation. The insets on right corresponding to the simulated crystal structures. Note: although there are high similarity, the

calculated structures are based on guest-free **TAMC**, while the experimental structures may still contain corresponding solvents.

6.2 Selective uptake from an EA-EtOH mixture in *α*-TAMC and TAMC polymorphs

For each mixture vapor-phase experiment, an open 5 mL vial containing 5.5 mg of *α*-**TAMC** or **TAMC** polymorph activated at 70 °C was placed in a sealed 20 mL vial containing 0.5 mL of EA and EtOH, respectively. Uptake in the **TAMC** polymorph crystals was measured by completely dissolving the crystals in $CDCl_3$ and measuring the ratio of EA or EtOH to **TAMC** by ^{1}H NMR, respectively

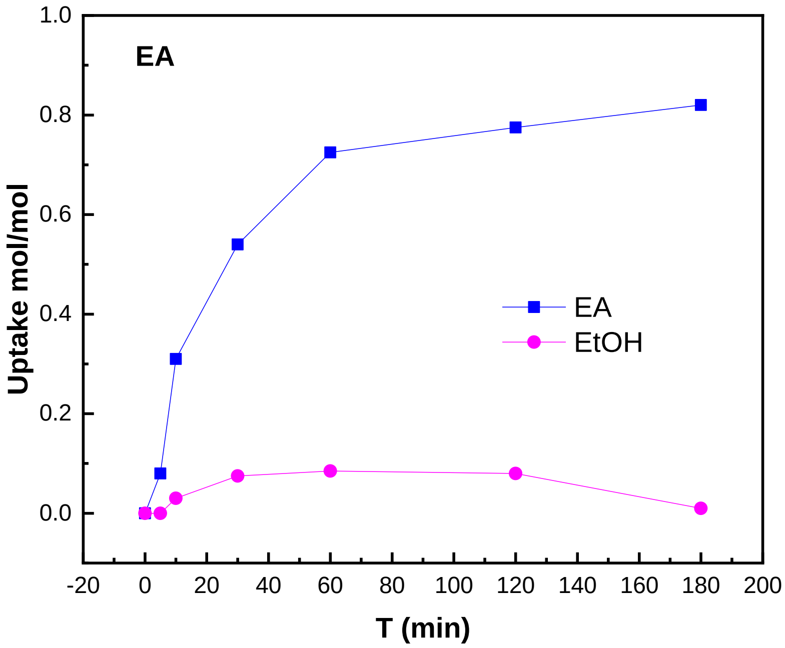

Figure S22. Time-dependent *α*-**TAMC** solid induced from EA sorption plot for EA/EtOH mixture vapor at 298 K.

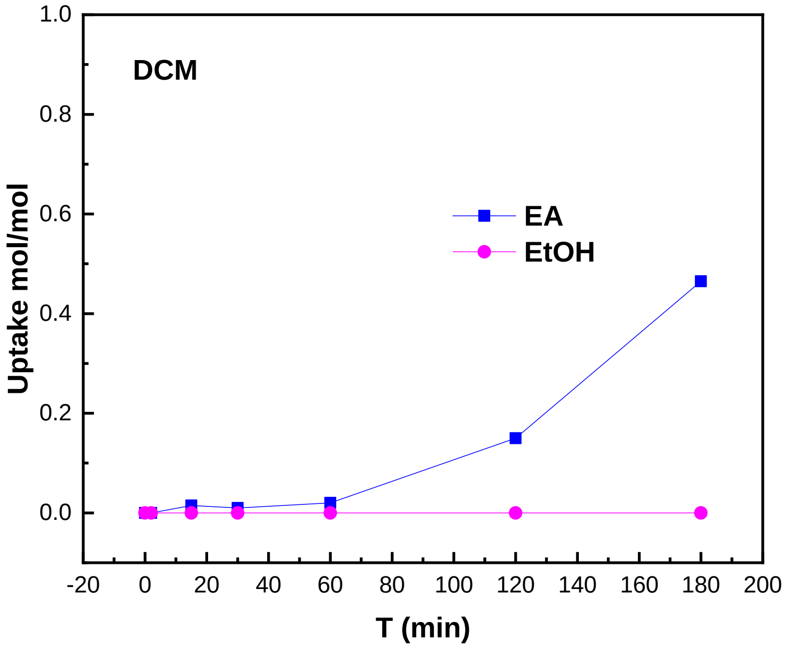

Figure S23. Time-dependent vapor sorption plot for EA/EtOH mixture vapor at 298 K for the **TAMC** polymorph induced by using **DCM** – note that the kinetics are slow compared to α-**TAMC**.

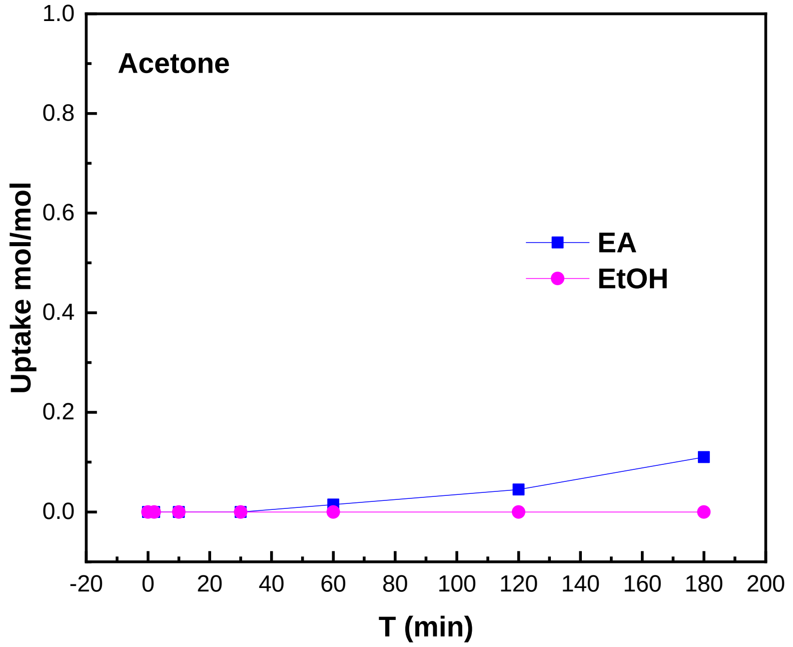

Figure S24. Time-dependent vapor sorption plot for EA/EtOH mixture vapor at 298 K in the **TAMC** polymorph induced from **acetone** – note that the kinetics are very slow compared to *α*-**TAMC**.

7. Breakthrough experiments

Breakthrough curves were measured for a fixed bed of *α*-**TAMC** at 298 K using an ABR automated breakthrough analyzer (manufactured by *Hiden Isochema*, Warrington, U.K.). *α*-**TAMC** for the breakthrough experiments was prepared in a similar manner to as described in the section of **1.2**. The *α*-**TAMC** powder activated at 70 °C was packed in a column made of a stainless steel (20 mL) between two layers of quartz wool and two layers of spherical inert glass beads (d = 2–3 mm) to improve the distribution of the inlet fluid. The gases were introduced through the bottom inlet of the adsorption bed. Frit gaskets installed at both the top and bottom ends of the adsorption bed were used to further prevent any potential powder contamination of the pipelines. All gases used were high purity. The gas lines were purged with the correct gas mixture before each experiment.

The EA-EtOH vapor mixture was produced by adding liquid mixture with ratio of 50:50 v/v into vapor generator and letting them reach gas-liquid equilibrium at 273 K and one bar pressure. The carrier gas for the vapors was N_2. The flow rate of each gas was controlled by

individual mass flow controllers. The system was controlled by the software *HIsorp* supplied by *Hiden Isochema*.

The m/z values used for detecting the gases were 28 for N_2 and 4 for He, the vapor were 31 for EtOH and 61 for EA. The reason for not using the base peak of EA is that m/z 43 overlapped signal of EtOH. It is known that the relative ratios of m/z 61 for EA is 14.9 % and the m/z 31 for EtOH is base peak with relative ratios of 100%. Therefore, the normalization and smoothing process were used in analysing raw signal of EA and EtOH because the intensity of EtOH signal will be much higher than EA signal with the same concentration.

The samples are activated in situ by heating and flowing helium through the column. This involved heating **TAMC** to 343 K for 12 hours. The vapors of interest were desorbed from the column by flowing helium through at the same rate as the vapors in the corresponding breakthrough experiment. The effluents were measured by an in-line mass spectrometer (Hiden DSMS, integrated with the breakthrough analyzer).

8. Computational details:

8.1 Conformational search

Conformers were generated using the mixed torsional/low-mode searching method implemented in the Maestro [12] software with a maximum of 10000 steps allowed. Energies during the initial search were calculated using the OPLS2005 force field[13] For identification of duplicates, conformers with an RMSD of greater than 0.3 Å were retained and all unique conformers with an energy less than 50 kJ mol^{-1} above the lowest energy structure were kept. Each unique conformer was re-optimized using density functional theory (DFT) with the B3LYP[14] functional and 6-311G** basis set with the D3 version of Grimme's dispersion correction with Becke-Johnson damping (GD3BJ)[15]; these calculations were performed using Gaussian09.[16] Redundant conformers after re-optimization with an all-atom RMSD < 0.3 Å were eliminated.

Eight unique conformers were located over a final energy range of 44 kJ/mol (Figure S25). The lowest energy conformer was separated from the rest by 18 kJ/mol, so this lowest energy conformer was used as a starting point for crystal structure prediction. The others were deemed to high in energy to be likely to produce low energy crystal structures.

8.2 CSP method

In the first stages of crystal structure prediction (CSP), molecules are held rigid. Crystal structures were generated starting with the lowest energy DFT-optimized conformer (section 8.1) and, to examine the effect of molecular geometry, CSP for EA:**TAMC** was also performed using the molecular geometries taken from the experimental crystal structures. Trial crystal structures were generated with one formula unit (**TAMC** for CSP of the host, and 1 EA + 1 **TAMC** for the solvate) in the asymmetric unit in the most commonly observed Sohncke space groups for organic molecules. Calculations for 1:1 EA:**TAMC** were performed in 6 space groups (P1, $P2_12_12_1$, $P2_12_12$, $P2_1$, C2 and $P4_12_12$) and extended to 13 space groups (P1; $P3_1$; $P3_2$; $P6_1$; $P6_5$; $P2_12_12_1$; $P2_12_12$; $P2_1$; C2; $P4_1$; $P4_3$; $P4_12_12$; $P4_32_12$) for the gas phase molecular geometry. CSP for pure (unsolvated) **TAMC** were performed using the gas phase optimized molecular geometry in 13 space groups (P1; $P3_1$; $P3_2$; $P6_1$; $P6_5$; $P2_12_12_1$; $P2_12_12$; $P2_1$; C2; $P4_1$; $P4_3$; $P4_12_12$; $P4_32_12$).

CSP was performed using a quasi-random sampling procedure, as implemented in the Global Lattice Energy Explorer software.[17] The generation of structures involves a low-discrepancy sampling of all structural variables within each space group: unit cell lengths and angles; molecular positions and orientations within the asymmetric unit. Space group symmetry was then applied and a geometric test was performed for overlap between molecules, which was removed by lattice expansion. The lattice energy minimization for each individual candidate crystal structure was performed using DMACRYS.[18] Intermolecular interactions were modelled using an empirically parametrized exp-6 repulsion-dispersion model and electrostatics described using atomic multipoles. The multipoles (up to hexadecapole on all atoms) were derived using GDMA[19] based on molecular charge densities obtained from B3LYP/6-311G** calculations on the single molecules, including multipoles up to hexadecapole on all atoms. Atom–atom repulsion and dispersion interactions were modelled using the FIT[20] intermolecular potential. Charge–charge, charge–dipole and dipole–dipole interactions were calculated using Ewald summation; all other intermolecular interactions were summed to a 25-Å cut-off between molecular centres-of-mass. All accepted trial structures were lattice energy minimized and the search was run until a set total number of lattice energy minimizations had been performed in each space group. For calculations performed with the gas phase optimised molecular geometry, 10,000 lattice energy minimizations were performed in each space group for pure **TAMC** and 20,000 lattice energy minimizations were performed per space group for 1:1 EA:**TAMC**. For EA:**TAMC** calculations using molecular geometries

from the experimentally determined crystal structures, these numbers were halved (10,000 minimizations per space group).

8.3 Periodic DFT re-optimization

Low energy crystal structures from CSP were re-optimized using solid state DFT to allow for relaxation of the molecular geometry in each predicted crystal structure. For pure **TAMC** crystal structures, we re-optimized all predicted crystal structures found within 20 kJ/mol of the global energy minimum from the rigid-molecule, force field predictions. For 1:1 EA:**TAMC**, we found that the position of EA within the crystal structures was very sensitive to the initial geometry of **TAMC**. Therefore, DFT re-optimization was performed on the low energy CSP structures from both sets of predictions (using the gas phase optimized **TAMC** geometry and the **TAMC** geometry taken from the experimental EA:**TAMC** crystal structure).

Re-optimization was performed in three steps. First, an optimization was performed with the unit cell parameters held fixed at those from the force field CSP structure, allowing atom positions in the unit cell to vary. This was followed by optimization including the unit cell parameters and a correction for the finite plane wave basis set. These first two steps used the PBE functional with pairwise TS dispersion correction,[21] ultrasoft pseodopotentials and a 500 eV plane wave cutoff on the basis set.

The final energies were calculated with a single point energy evaluation with the basis set increased to a 750 eV energy cutoff and the TS dispersion correction replaced by the many-body MBD@ rsSCS dispersion model.[22] A maximum k-point separation of 0.04 Å^{-1} was used in all calculations.

These calculations were performed using CASTEP verstion 17.21.[23]

8.4 Substructure search

For each predicted crystal structure, the corresponding position of EA and **TAMC** are identified using the CSD Python Application Programming Interface, together with in-house scripts. If the distance between the carbon atom on the methyl/ethyl and the centroid of **TAMC** are less than 2.0 Å, the methyl/ethyl end of EA is considered as inside the **TAMC** cavity. Note that, for efficiency, the centroid of six nitrogen atoms on the **TAMC** molecule is used to represent the centroid of the whole **TAMC** molecule.

8.5 Ideal adsorbed solution theory (IAST)

Mixture adsorption equilibria were predicted by ideal adsorbed solution theory (IAST)[24] using single-component adsorption data, measured experimentally. A detailed description of the approach used to obtain the results reported here can be found in the literature.[25] To apply IAST, single-component adsorption isotherms were specified by fitting an isotherm equation to the discrete, experimental adsorption measurements.

8.6 ESP and NCI analysis

The analysis of ESP and NCI are undertaken by the Multiwfn program,[26] employing the wave functions generated with B3LYP/6-311g(d,p) and visualized through the VMD package.[27] The NCI is based on single point calculate at the experiment geometry of EA@**TAMC**. The geometry of EA and EtOH for ESP analyse are optimized by *Gaussian*16.

8.7 Interaction energy calculation

Periodic DFT calculations were carried out with the Vienna ab initio Simulation Package (VASP) version 5.4.4.[28] The projector augmented-wave (PAW) method was applied to describe the electron-ion interactions.[29] Generalized gradient approximation (GGA) with the Perdew–Burke–Ernzerhof (PBE) exchange-correlation functional was adopted to treat electron interaction energy.[30] Grimme's semi-empirical DFT-D3[31] scheme was used here to give a better description of long range interactions; the latest Becke–Johnson damping functions[15, 32] for the DFT-D3 method were adopted. A kinetic-energy cutoff of 500 eV was used to define the plane-wave basis set. During geometry optimizations, the Hellmann–Feynman force convergence criterion on each atom was set to smaller than 0.01 eV/Å Convergence threshold of self-consistency was set to 10^{-5} eV in total energy. Gamma-centered K-point meshes were calculated by VASP for each structure using a gamma-centered k-spacing of 0.2 $Å^{-1}$. The geometry of all structures used here are after optimized. The interaction energy are evaluated via the fllowing equation:

$$E_{int} = (E_{X@\mathbf{TAMC}}-E_{alpha\text{-}\mathbf{TAMC}}-2*E_X)/2 \qquad (1)$$

Where X represent the EA or EtOH.

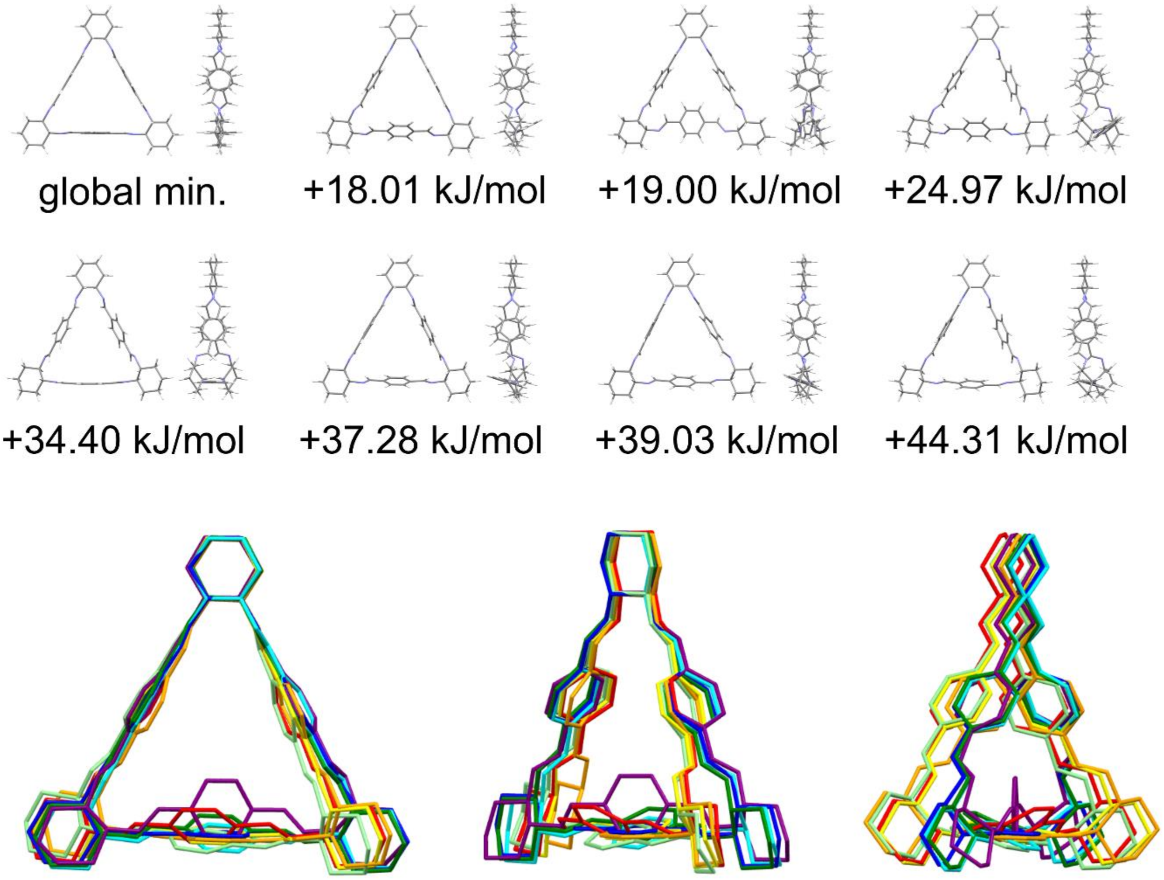

Figure S25. Unique conformers for the isolated **TAMC** molecule. The top two rows show the 8 unique conformers after DFT re-optimization, showing two views per conformer. Energies are relative to the lowest energy conformer, taken from the DFT calculation. The bottom row shows an overlay of the 8 conformers from three views.

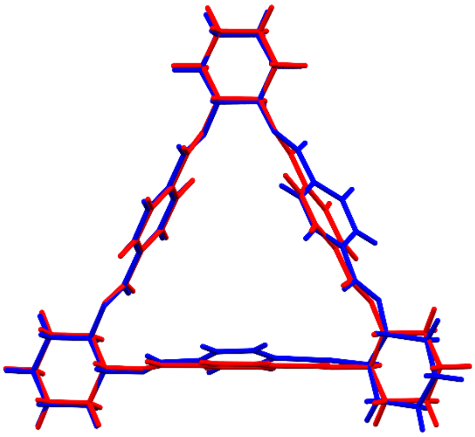

Figure S26. Difference in molecular geometry of gas phase minimum conformer (red) and the conformer from experiment EA@**TAMC** structure (blue).

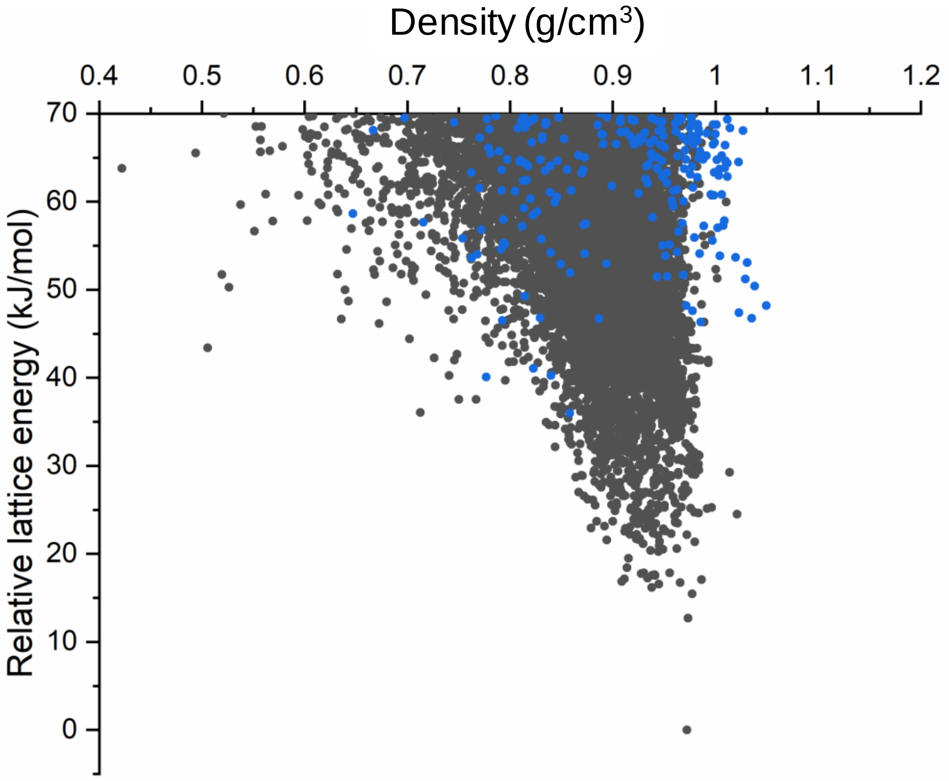

Figure S27. CSP landscape for a 1:1 composition of EA:**TAMC** using the gas phase minimum conformer with a 70 kJ/mol relative lattice energy window and 13 Sohncke space groups. Each point here corresponds to a predicted crystal structure that is a local minimum on the lattice energy surface. The colour-coded is given by the positioning of the EA molecule relative to the **TAMC** cavity. Blue dots for the ethyl end of EA inside **TAMC** and black dots for EA outside **TAMC**. There are no predicted structures with the methyl end of EA inside **TAMC**.

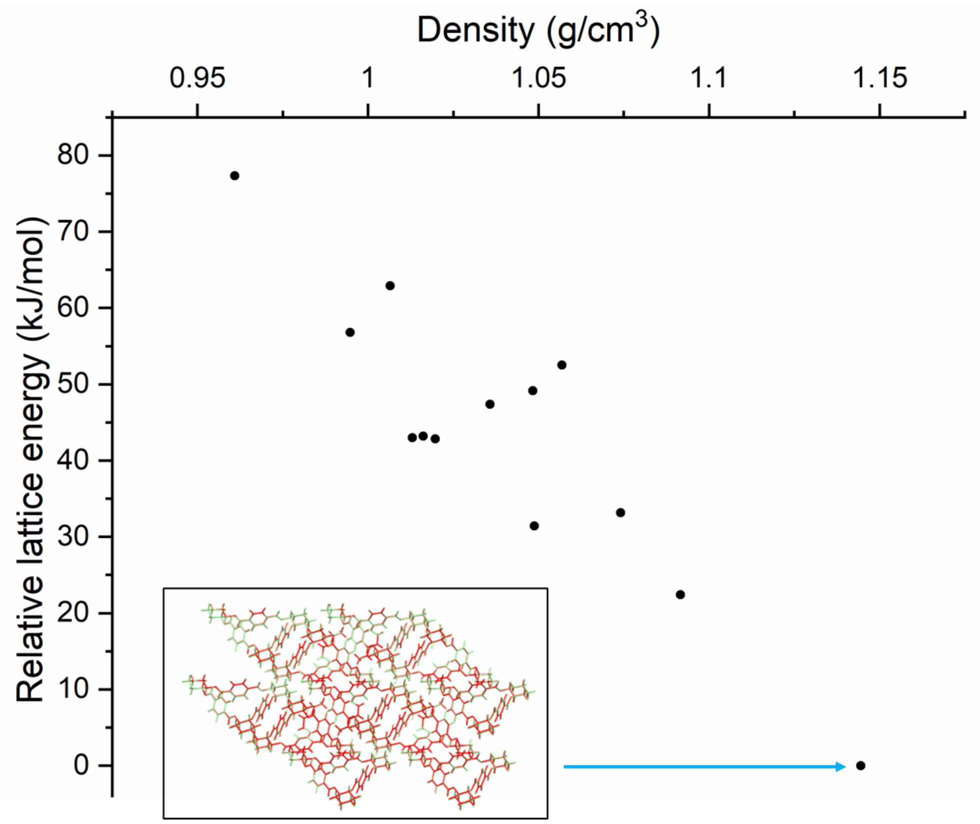

Figure S28. CSP landscape for a 1:1 composition of EA:**TAMC** after density functional theory (DFT) re-optimization with no geometry restrict was given. The origin structures were selected out from the lowest 20kJ/mol window of CSP with gas phase. Each point here corresponds to a predicted crystal structure that is a local minimum on the DFT lattice energy surface. The experimental result is predicted as the global minimal packing structure. The inset here are crystal packing similarity matching result of experimental structure (red) and simulated structure (green).

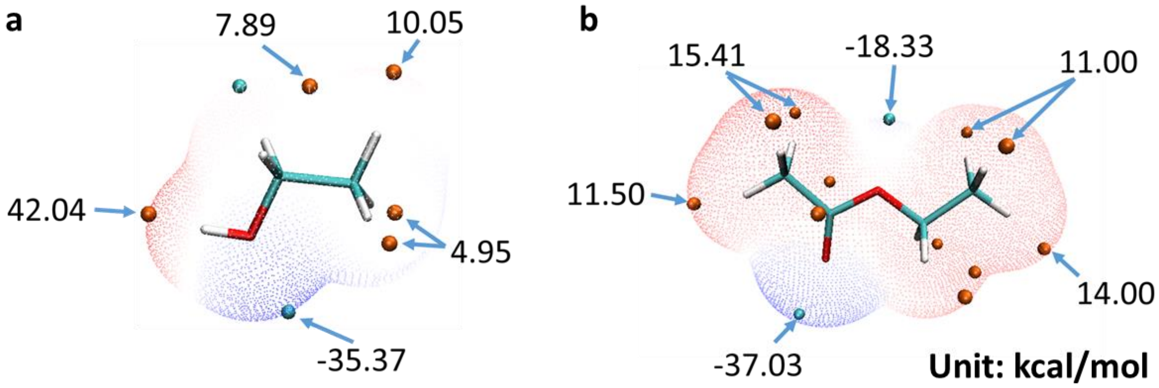

Figure S29. Electrostatic surface potential (ESP) of the (**a**): EA; (**b**): EtOH, blue point represents most negative ESP ($V_{s,min}$), and red point represents most positive ESP ($V_{s,max}$). Values are given in kcal/mol.

The $V_{s,max}$ appear along the extension of the C-H bonds. In contrast, the C=O in the ester group shows most negative **ESP**, attributed to the unpaired electrons on this atom. EA with these extreme point sites interacting with **TAMC** could form stable co-crystal.

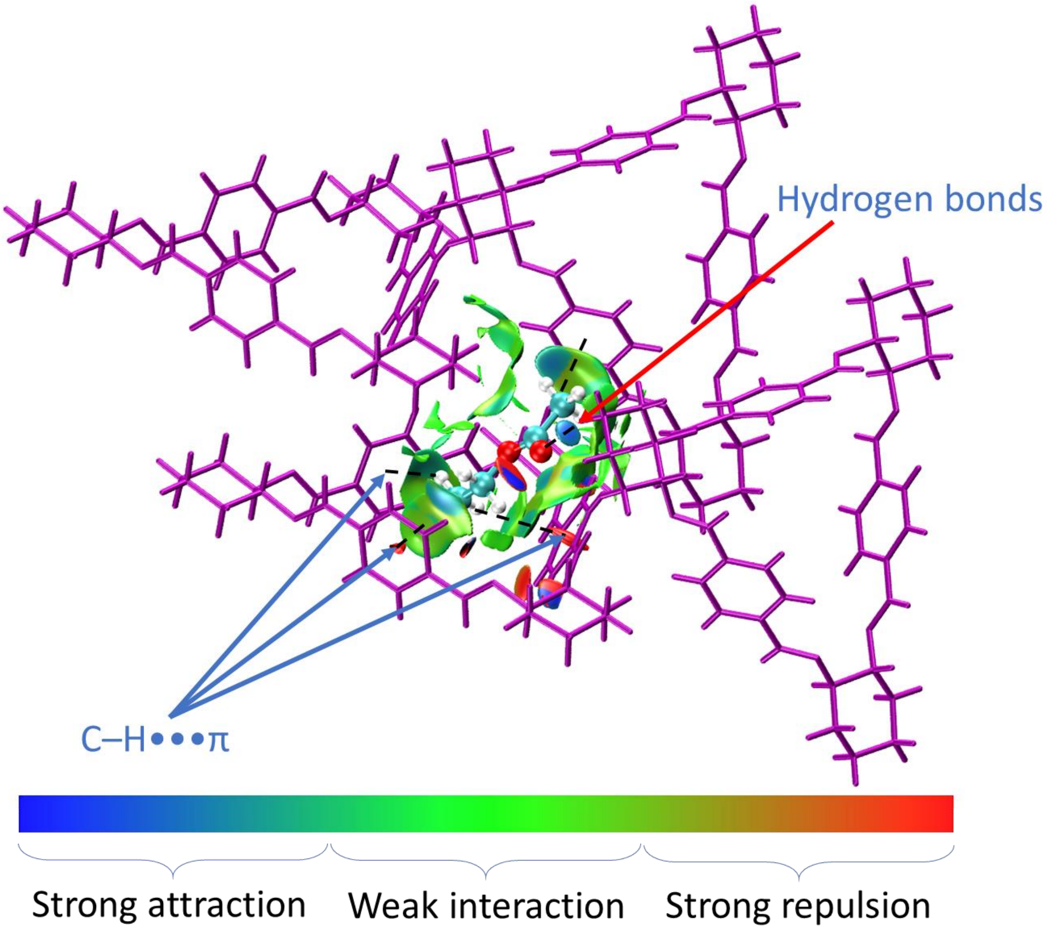

Figure S30. Non-covalent interactions index isosurface of EA@**TAMC.**

References

[1] N. Kuhnert, D. Marsh, D. C. Nicolau, *Tetrahedron: Asymmetry* **2007**, *18*, 1648.

[2] a) A. A. Coelho, *J. Appl. Crystallogr.* **2018**, *51*, 210; b) A. Coelho, TOPAS-Academic, v5, Coelho Software: Brisbane, Australia, **2012**.

[3] a) L. Krause, R. Herbst-Irmer, G. M. Sheldrick, D. Stalke, *J. Appl. Crystallogr.* **2015**, *48*, 3; b) G. Sheldrick, SADABS, version 2008/1; University of Göttingen: Göttingen, Germany, **2008**.

[4] G. Winter, D. G. Waterman, J. M. Parkhurst, A. S. Brewster, R. J. Gildea, M. Gerstel, L. Fuentes-Montero, M. Vollmar, T. Michels-Clark, I. D. Young, N. K. Sauter, G. Evans, *Acta Crystallogr D Struct Biol* **2018**, *74*, 85.

[5] G. M. Sheldrick, *Acta Crystallogr. A* **2015**, *71*, 3.

[6] G. M. Sheldrick, *Acta Crystallogr C Struct Chem* **2015**, *71*, 3.

[7] O. V. Dolomanov, L. J. Bourhis, R. J. Gildea, J. A. K. Howard, H. Puschmann, *J. Appl. Crystallogr.* **2009**, *42*, 339.

[8] J. Gawroński, H. Kołbon, M. Kwit, A. Katrusiak, *J. Org. Chem.* **2000**, *65*, 5768.

[9] J. Gawronski, H. Kolbon, M. Kwit, A. Katrusiak, *J. Org. Chem.* **2000**, *65*, 5768.

[10] A. Janiak, C. Esterhuysen, L. J. Barbour, *Chem. Commun.* **2018**, *54*, 3727.

[11] M. Thommes, K. Kaneko, A. V. Neimark, J. P. Olivier, F. Rodriguez-Reinoso, J. Rouquerol, K. S. W. Sing, *Pure Appl. Chem.* **2015**, *87*, 1051.

[12] Schrödinger, *Schrödinger: New York*, **2018**.

[13] J. L. Banks, H. S. Beard, Y. Cao, A. E. Cho, W. Damm, R. Farid, A. K. Felts, T. A. Halgren, D. T. Mainz, J. R. Maple, R. Murphy, D. M. Philipp, M. P. Repasky, L. Y. Zhang, B. J. Berne, R. A. Friesner, E. Gallicchio, R. M. Levy, *J. Comput. Chem.* **2005**, *26*, 1752.

[14] a) A. D. Becke, *J. Chem. Phys.* **1993**, *98*, 5648; b) C. T. Lee, W. T. Yang, R. G. Parr, *Phys. Rev. B* **1988**, *37*, 785.

[15] S. Grimme, S. Ehrlich, L. Goerigk, *J. Comput. Chem.* **2011**, *32*, 1456.

[16] M. Frisch, G. Trucks, H. Schlegel, G. Scuseria, M. Robb, J. Cheeseman, G. Scalmani, V. Barone, G. Petersson, H. Nakatsuji, *Wallingford CT*.

[17] D. H. Case, J. E. Campbell, P. J. Bygrave, G. M. Day, *J. Chem. Theory Comput.* **2016**, *12*, 910.

[18] S. L. Price, M. Leslie, G. W. Welch, M. Habgood, L. S. Price, P. G. Karamertzanis, G. M. Day, *PCCP* **2010**, *12*, 8478.

[19] A. Stone, *University of Cambridge* **2010**.

[20] D. S. Coombes, S. L. Price, D. J. Willock, M. Leslie, *J. Phys. Chem.* **1996**, *100*, 7352.

[21] A. Tkatchenko, M. Scheffler, *Phys. Rev. Lett.* **2009**, *102*, 073005.

[22] A. Ambrosetti, A. M. Reilly, R. A. DiStasio, Jr., A. Tkatchenko, *J. Chem. Phys.* **2014**, *140*, 18A508.

[23] S. J. Clark, M. D. Segall, C. J. Pickard, P. J. Hasnip, M. I. Probert, K. Refson, M. C. Payne, *Z. Kristallogr. Cryst. Mater.* **2005**, *220*, 567.

[24] D. W. Hand, S. Loper, M. Ari, J. C. Crittenden, *Environ. Sci. Technol.* **1985**, *19*, 1037.

[25] N. F. Cessford, N. A. Seaton, T. Düren, *Ind. Eng. Chem. Res.* **2012**, *51*, 4911.

[26] T. Lu, F. Chen, *J. Comput. Chem.* **2012**, *33*, 580.

[27] W. Humphrey, A. Dalke, K. Schulten, *J. Mol. Graphics* **1996**, *14*, 33.

[28] a) G. Kresse, J. Furthmüller, *Phys. Rev. B* **1996**, *54*, 11169; b) G. Kresse, J. Furthmüller, *Comput. Mater. Sci.* **1996**, *6*, 15.

[29] G. Kresse, D. Joubert, *Phys. Rev. B* **1999**, *59*, 1758.

[30] a) J. P. Perdew, K. Burke, M. Ernzerhof, *Phys. Rev. Lett.* **1996**, *77*, 3865; b) Y. Zhang, W. Yang, *Phys. Rev. Lett.* **1998**, *80*, 890.

[31] S. Grimme, J. Antony, S. Ehrlich, H. Krieg, *J. Chem. Phys.* **2010**, *132*, 154104.

[32] A. D. Becke, E. R. Johnson, *J. Chem. Phys.* **2005**, *122*, 154104.
